# Advanced Thermal Interface Materials: Insights into Low‐Temperature Sintering and High Thermal Conductivity of MgO

**DOI:** 10.1002/adma.202510237

**Published:** 2025-08-27

**Authors:** Su‐Jin Ha, Hye‐Jeong Jang, Hui‐jin Son, Young Kook Moon, Hyun‐Ae Cha, Jong‐Jin Choi, Jee‐Hyuk Ahn, Byung‐Dong Hahn, Kyung‐Hoon Cho, Do‐Cheon Ahn, Jun Lim, Sang‐Chae Jeon, In Chul Jung, Youngsup Song, Hao Zhou, Tianli Feng, Cheol‐Woo Ahn

**Affiliations:** ^1^ Functional Ceramics Department Powder & Ceramics Division Korea Institute of Materials Science (KIMS) Changwon Gyeongnam 641‐831 Republic of Korea; ^2^ School of Materials Science and Engineering Kumoh National Institute of Technology 61 Daehak‐ro Gumi Gyeongbuk 39177 Republic of Korea; ^3^ Pohang Accelerator Laboratory Pohang University of Science and Technology 127 Jigokro Pohang Kyungbuk 37637 Republic of Korea; ^4^ School of Materials Science and Engineering Changwon National University 20 Changwondaehak‐ro Changwon Gyeongsangnam‐do 51140 Republic of Korea; ^5^ Soulmaterial 27 Sampung‐ro Gyeongsan‐si Gyeongsangbuk‐do 38541 Republic of Korea; ^6^ Department of Mechanical and Aerospace Engineering University of Florida Gainesville FL 32611 USA; ^7^ Department of Mechanical Engineering University of Utah 1495 East 100 South Salt Lake City Utah 84112 USA

**Keywords:** battery package, electric vehicle, heat‐dissipation material, magnesia, thermal Interface material

## Abstract

The escalating frequency of electric vehicle (EV) fires has underscored the critical importance of effective thermal‐management in battery package (TMBP). A key challenge in current TMBP lies with the low thermal conductivity (TC, 3 W m^−1^ K^−1^) of commercial alumina‐polymer composite (thermal interface materials, TIM). While magnesia (MgO) TIMs, which show high TC (8–10 W m^−1^ K^−1^, this study) and low cost, are emerging as an alternative heat‐dissipation material (HDM), their full potential remains untapped. Here, the development of novel MgO (≥ 80 W m^−1^ K^−1^) and MgO TIMs is presented as next‐generation HDMs, designed to outperform conventional alumina (20–30 W m^−1^ K^−1^) and alumina TIMs. Crucially, the fundamental mechanisms enabling our new MgO to achieve an unprecedented TC of ≥ 80 W m^−1^ K^−1^ are elucidated, significantly surpassing the previously reported range of 40–60 W m^−1^ K^−1^. This study provides fundamental insights into achieving such high thermal conductivity in MgO. Furthermore, it is demonstrated that this novel MgO TIM cools EV batteries three times faster than commercial alternatives, offering a robust solution for effective EV fire prevention. Consequently, this high‐TC MgO is poised to contribute significantly to enhancing EV safety.

## Graphical abstract

MgO has emerged as a promising candidate for improving thermal management in battery systems owing to its high thermal conductivity. In this study, it is demonstrate that the thermal conductivity of MgO is historically underestimated due to previously overlooked material behaviors. Furthermore, MgO‐based TIMs can make a significant contribution to EV safety due to their excellent heat dissipation properties.

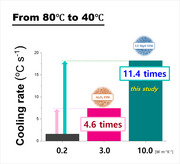

## 1 Introduction

The escalating concerns for the global environment are driving a swift transition toward the era of electric vehicles (EVs).^[^ ^1^, ^2^, ^3^, ^4^ ^]^ However, the frequent occurrence of EV fires, primarily due to battery overheating, poses a significant safety challenge and a major obstacle to this transition.^[^ ^5^, ^6^, ^7^, ^8^, ^9^ ^]^ While all‐solid‐state batteries are being extensively researched as a long‐term solution, their commercialization remains distant.^[^ ^10^, ^11^, ^12^, ^13^, ^14^, ^15^ ^]^ Consequently, enhancing the safety of currently employed commercial battery packages (BPs) in EVs is paramount. Here, we report a significant discovery in thermal management materials, an essential component for achieving superior safety in EV battery packages.

EV safety critically depends on the thermal conductivity (TC) of the thermal interface material (TIM), which efficiently dissipates heat generated by the battery.^[^ ^16^, ^17^, ^18^, ^19^ ^]^Specifically, TIMs are essential for cooling the battery by transferring heat from the battery cells to BP case.^[^ ^19^, ^20^, ^21^, ^22^, ^23^ ^]^ Thus, they require fabrication from materials possessing high TC. Currently, commercial TIMs are predominantly alumina‐based, offering competitive price but limited TC (≈20–30 W m^−1^ K^−1^).^[^ ^24^, ^25^, ^26^ ^]^ While nitrides such as AlN, Si_3_N_4_, and BN exhibit excellent TCs (≥ 70 W m^−1^ K^−1^), their high cost precludes them as viable substitutes for alumina.^[^ ^27^, ^28^, ^29^ ^]^

Magnesia (MgO), priced comparably to alumina, is widely recognized as a viable replacement for alumina due to its TC ranging from 40 to 60 W m^−1^ K^−1^, approximately twice that of alumina.^[^ ^30^, ^31^ ^]^ This TC range for MgO has been broadly accepted, aligning closely with the theoretically calculated TC of MgO single crystals (50–60 W m^−1^ K^−1^), reinforcing its perceived thermal performance.^[^ ^32^, ^33^, ^34^, ^35^, ^36^, ^37^, ^38^ ^]^ Moreover, the thermal conductivities of MgO materials prepared by various methods continue to be reported in recent studies as summarized at Table S1 (Supporting Information).^[^ ^30^, ^31^, ^39^, ^40^, ^41^, ^42^, ^43^, ^44^, ^45^, ^46^, ^47^, ^48^ ^]^ Consequently, MgO has been considered to possess inherently lower TC than nitride materials, which typically exhibit TCs of ≥ 70 W m^−1^ K^−1^.

In this study, however, we challenge this prevailing understanding, demonstrating that the intrinsic TC of MgO has been significantly underestimated. This study aims to demonstrate that the dopant concentrations previously employed for the densification of MgO have been excessively high. As a result, we reveal that MgO can achieve a high TC of ≥ 80 W m^−1^ K^−1^, a value competitive with, and not far behind, that of nitrides.

Furthermore, this manuscript showcases the superior heat dissipation performance of MgO‐based TIMs. Specifically, we delineate why MgO TIMs are more appropriate for the thermal management of BPs and enhancing EV safety. Indeed, leading companies are currently evaluating MgO TIMs as a replacement for alumina TIMs, underscoring their substantial potential to contribute to EV safety.

## 2 Results and Discussion

### 2.1 High Thermal‐Conductity Demanded for EV Safety

**Figure 1:**
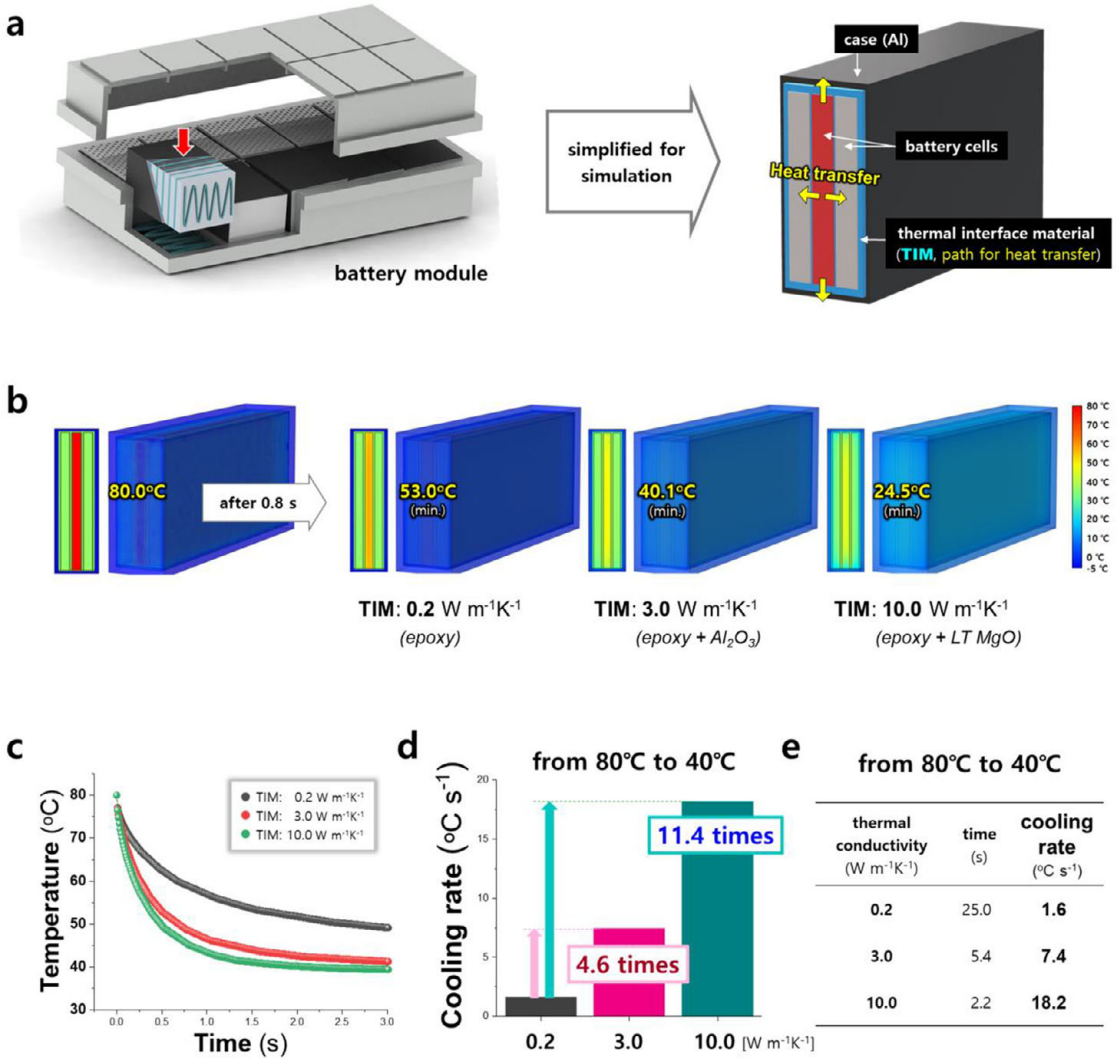
Enhanced thermal management in battery modules using TIMs composed of low‐temperature sintered MgO. a) Schematic illustration of battery module architecture and corresponding simplified thermal simulation model used to evaluate TIM performance. b) Simulated temperature distribution across battery cell after 0.8 s from initial state at 80 °C, using TIMs with varying thermal conductivities (0.2, 3.0, and 10.0 W m^−1^ K^−1^). c) Temporal evolution of cell temperature under identical initial conditions (80 °C) for modules incorporating TIMs with different thermal conductivities. d,e) Comparative analysis of time required and effective cooling rates for cell temperature to decrease from 80 to 40 °C, highlighting the impact of TIM thermal conductivity on thermal dissipation efficiency.

As depicted in **Figure** **1**a, a BP comprises battery cells, TIM, and an aluminum (Al) case. Heat generated by the battery cell is transferred through the TIM to the Al case (237 W m^−1^ K^−1^),^[^ ^49^, ^50^ ^]^ which is then cooled by water or air. Lithium‐ion batteries typically operate within 30–40 °C, while 80 °C is considered a dangerous temperature, as explained at Table S2 (Supporting Information). Assuming a sudden temperature spike to 80 °C in a battery cell, a TIM with a TC of 10 W m^−1^ K^−1^ demonstrated superior cooling efficacy compared to a battery module utilizing a commercial TIM of 3.0 W m^−1^ K^−1^ (Figure 1b–e). Specifically, after 0.8 s, the cell with the 10.0 W m^−1^ K^−1^ TIM cooled more effectively to 24.5 °C, whereas the commercial cell with the 3.0 W m^−1^ K^−1^ TIM only cooled to 40.1 °C, exceeding the normal operating temperature, as shown in Figure 1b. More precisely, Figure 1c illustrates that the battery cell temperature decreased significantly more effectively over time in a BP incorporating a 10.0 W m^−1^ K^−1^ TIM (green color) compared to a 3.0 W m^−1^ K^−1^ TIM. Notably, Figure 1d,e highlights that the cooling rate from 80 to 40 °C is 2.5 times faster in the cell employing the 10.0 W m^−1^ K^−1^ TIM than in the one with the 3.0 W m^−1^ K^−1^ TIM. Given that the safe operating temperature for lithium‐ion batteries in electric vehicles is generally 30–40 °C, replacing a 3.0 W m^−1^ K^−1^ TIM with a 10.0 W m^−1^ K^−1^ TIM can efficiently prevent EV fires by substantially improving the cooling performance of the BP.

TIMs are fabricated by dispersing ceramic fillers within a polymer. The polymer component is primarily responsible for adhesion, while the ceramic filler facilitates heat transfer. Therefore, utilizing ceramic materials with intrinsically higher TC is crucial for enhancing the overall TC of the TIM. In this study, we successfully engineered an advanced MgO TIM demonstrating a TC of 10.0 W m^−1^ K^−1^, representing a significant improvement over the 3.0 W m^−1^ K^−1^ of commercial alumina TIMs. This advancement was achieved by incorporating a newly developed MgO (MgO sintered at low temperature, LT MgO). This material exhibits an unprecedented TC of ≥ 80 W m^−1^ K^−1^, which is markedly higher than the 20–30 W m^−1^ K^−1^ typically associated with alumina. While the TC of MgO is conventionally reported to be in the range of 40–60 W m^−1^ K^−1^, the LT MgO exhibits a superior TC of ≥ 80 W m^−1^ K^−1^.

### 2.2 Fundamental Study of Sintering Behavior in MgO

**Figure 2:**
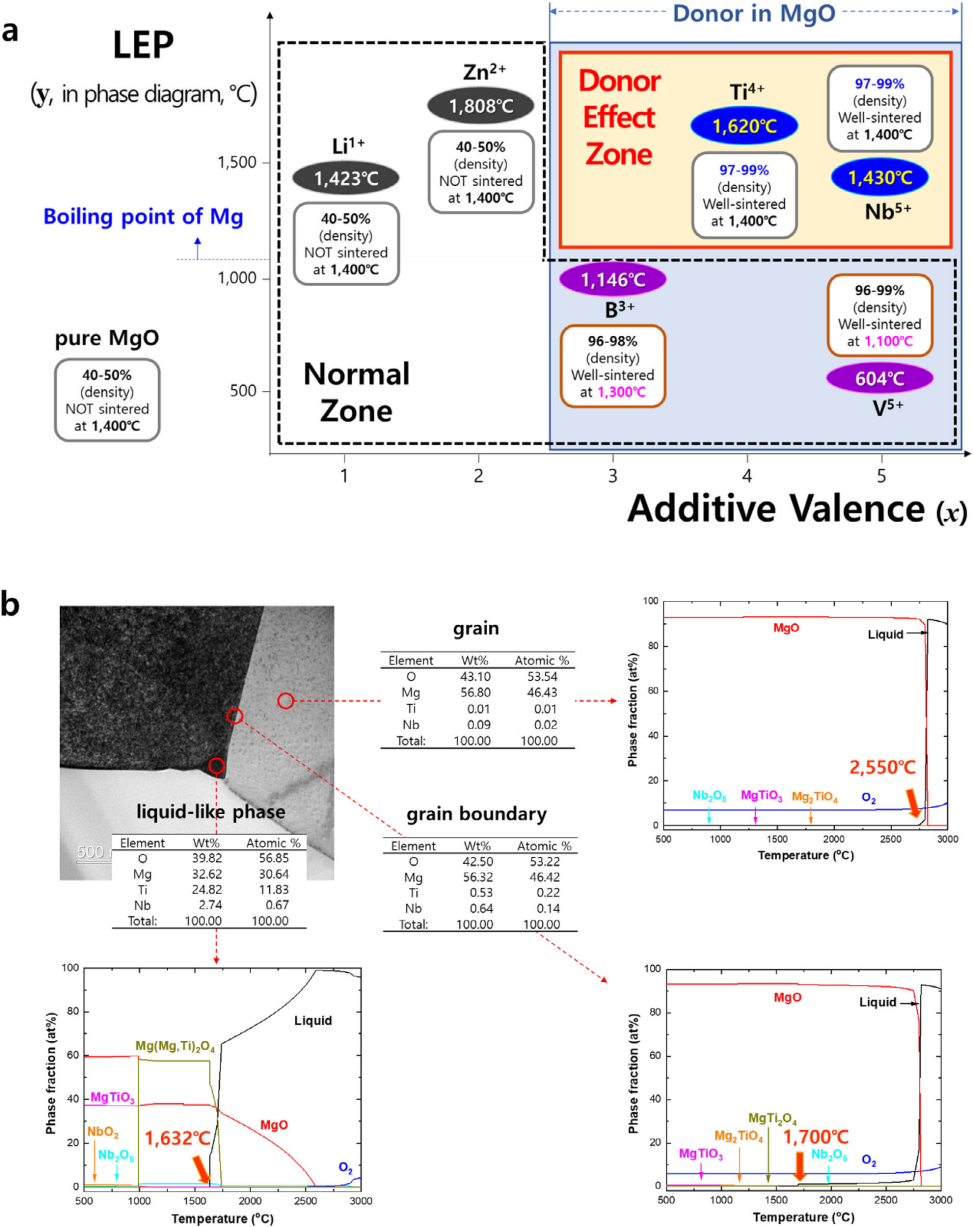
Donor effect zone sintered at 1400 °C and phase diagrams of grain, grain boundary, and liquid‐like phase in d‐MgO (donors: Ti⁴⁺ and Nb⁵⁺). a) Normal zone and donor effect zone exhibiting distinct sintering behavior. b) Phase diagrams generated using FactSage 8.1 (Thermfact/CRCT, Montreal, QC, Canada) with FactPS, FToxid, and FTlite databases. According to phase equilibria, a liquid‐like phase in MgO does not form below 1400 °C when Ti⁴⁺ or Nb⁵⁺ is incorporated. Liquid‐like phase identified via TEM analysis (Figure S4, Supporting Information).

For MgO to effectively replace commercial alumina, which is typically sintered at 1500–1600 °C, it is economically advantageous for MgO to be manufactured at a lower temperature. Initially, the various additives were incorporated into MgO to achieve sintering at 1400 °C, a temperature below 1500 °C. As shown in **Figure** **2**a, the *x*‐axis represents the additive valence (e.g., Li⁺: 1, Zn^2^⁺: 2, B^3^⁺: 3, Ti⁴⁺: 4, and Nb⁵⁺: 5), while the *y*‐axis indicates the lowest eutectic point (LEP) observed on the phase diagram of AO_w_‐MgO (where A represents metals such as Li, Zn, B, Ti, Nb, and V).

For instance, the Li_2_O‐MgO phase diagram shows an LEP of 1423 °C (Figure 2a; Figures S1 and S2, Supporting Information), whereas pure MgO typically requires sintering temperatures in the range of 1800–2000 °C. Consequently, Li‐added MgO (L‐MgO) specimens, when attempted to be sintered at 1400 °C, exhibited a low relative‐density of 40–50%, as depicted in Figure 2a and Figure S3 (Supporting Information). Zn‐added MgO (Z‐MgO), with an LEP of 1808 °C, displayed similar characteristics to L‐MgO. The microstructure of both L‐MgO and Z‐MgO sintered at 1400 °C closely resembled that of pure MgO sintered at the same temperature, as evident from the left SEM images in Figure S3 (Supporting Information).

In contrast, B‐ or V‐added MgO (B‐MgO or V‐MgO) exhibited significantly lower LEPs (1146 °C for B‐MgO and 604 °C for V‐MgO, as shown in Figure 2a and Figure S1, Supporting Information). This allowed B‐MgO and V‐MgO to be well‐sintered at 1300 and 1100 °C, respectively. These specimens achieved high densities of 96–99% and large grain sizes exceeding 10 µm, attributed to the formation of a liquid phase (Figure 2a; Figures S3 and S4, Supporting Information).

In our investigation into the sintering behavior of additive‐doped MgO, a distinct phenomenon was observed in donor‐doped MgO (d‐MgO). The LEPs for Ti‐ or Nb‐doped MgO (T‐MgO: 1620 °C; N‐MgO: 1430 °C) was both found to exceed 1400 °C. Accordingly, as shown in the phase diagrams in Figures S1 and S2 (Supporting Information), no liquid‐like phase (LP) is anticipated to form below 1400 °C in T‐MgO, N‐MgO, or in the co‐doped composition (TN‐MgO). Thermodynamic calculations performed using FactSage 8.1 (Thermfact/CRCT, Montreal, QC, Canada) further confirmed that the LP composition in TN‐MgO emerges only at temperatures ≥ 1632 °C (Figure 2b), and that LP formation is thermodynamically unfavorable below 1510 °C when Ti⁴⁺ and Nb⁵⁺ ions are introduced into the MgO matrix (Figure S5, Supporting Information). These results indicate that conventional phase diagrams alone are insufficient to predict LP formation in T‐MgO and N‐MgO.

Nevertheless, T‐MgO, N‐MgO, and TN‐MgO exhibited distinctly different sintering behavior compared to L‐MgO and Z‐MgO. When sintered at 1400 °C, these donor‐doped specimens achieved exceptionally high relative‐densities (97–99%) and exhibited substantial grain growth exceeding 10 µm, which is attributed to the LP formation, as illustrated in Figure 2a and Figures S3,S4 and S6 (Supporting Information). The region highlighted by the red box in Figure 2a delineates the so‐called “Donor Effect Zone”, where enhanced densification and grain coarsening are observed. This characteristic behavior is further corroborated by the data summarized in Table S3 (Supporting Information). Moreover, differential thermal analysis (DTA), presented in Figure S7 (Supporting Information), reveals endothermic events in the temperature range of 1100 to 1200 °C, providing additional evidence for LP formation in d‐MgO and suggesting the onset of incipient melting.

To distinguish between the Normal Zone and the Donor Effect Zone shown in Figure 2a, we determined the occupancy of Mg and O elements using Rietveld refinement. This refinement was performed on high‐resolution synchrotron powder X‐ray diffraction (XRD) data, acquired from the high‐resolution synchrotron powder X‐ray diffraction (HRPD) beamline at Pohang Light Source II (PLS‐II) in Korea, as indicated in Figure S8. Rietveld refinement allows for a relative comparison of the occupancy levels between Mg and O atoms across different specimens, enabling identification of which element is more deficient in each case. However, the absolute occupancy values obtained from this analysis lack quantitative significance and should not be interpreted as precise stoichiometric metrics.

This analysis revealed a deficiency of the Mg element in the MgO specimens, with this deficiency becoming more pronounced at higher manufacturing temperatures. Notably, the addition of donor elements exacerbated this deficiency. Therefore, it is plausible that the occupancy difference between O and Mg (Figure S8, Supporting Information) potentially increases in MgO when donors such as Ti⁴⁺ and Nb⁵⁺ are incorporated.

(1) $$\mathrm{Ti}O_{2}\underset{2\mathrm{MgO}}{\to }Ti_{\ddot{M}g}+{V^{\prime \prime }}_{\mathrm{Mg}}+20_{O}^{X}$$

(2) $$Nb_{2}O_{5}\underset{5\mathrm{MgO}}{\to }2Nb_{\ddot{M}g}+3\;{V^{\prime \prime }}_{\mathrm{Mg}}+50_{O}^{X}$$

TN‐MgO is MgO + 0.15 at.% Ti⁴⁺ + 0.10 at.% Nb⁵⁺; therefore, the generation of Mg vacancies induced by donor doping cannot exceed 0.3 at.% in principle. As shown in Figure 2b, a significant portion of the added donor species likely remains in the grain boundaries and the surface of the specimen as components of the liquid‐like phase, with only a minimal fraction incorporated within the grain interior. The localized concentrations of Ti⁴⁺ and Nb⁵⁺ ions detected inside the grains are estimated to be ≤ 0.03 at.%, as evidenced in Figure 2b.

Furthermore, the incorporation of donor species can promote the formation of secondary phases, and the extent of such phase formation tends to increase with higher dopant concentrations. As shown in Figure S9 (Supporting Information), excessive donor addition results in the precipitation of a substantial amount of secondary phases, which in turn suppresses grain growth. Therefore, the amount of donor dopant should be minimized to avoid undesirable microstructural effects. For TN‐MgO, secondary phase formation appears to be effectively suppressed, as indicated by the negligible presence of secondary phase peaks in the XRD data presented in Figure S8 (Supporting Information).

Figure S10a (Supporting Information) elucidates a fundamental experiment investigating the role of donors in the sintering behavior of d‐MgO. In this setup, the donor was strategically positioned along the centerline of the MgO specimen, and localized grain growth was observed specifically where the donor was present. Furthermore, the abnormally grown grain (AG) was evident in d‐MgO, as shown in Figure S10b (Supporting Information). These results clearly demonstrate that, in d‐MgO, LP can form at the surface of MgO particles due to the presence of donor species, which in turn promotes both grain growth and densification. The occurrence of AGs can be attributed to local differences in donor concentration within the sintered specimens.

**Figure 3:**
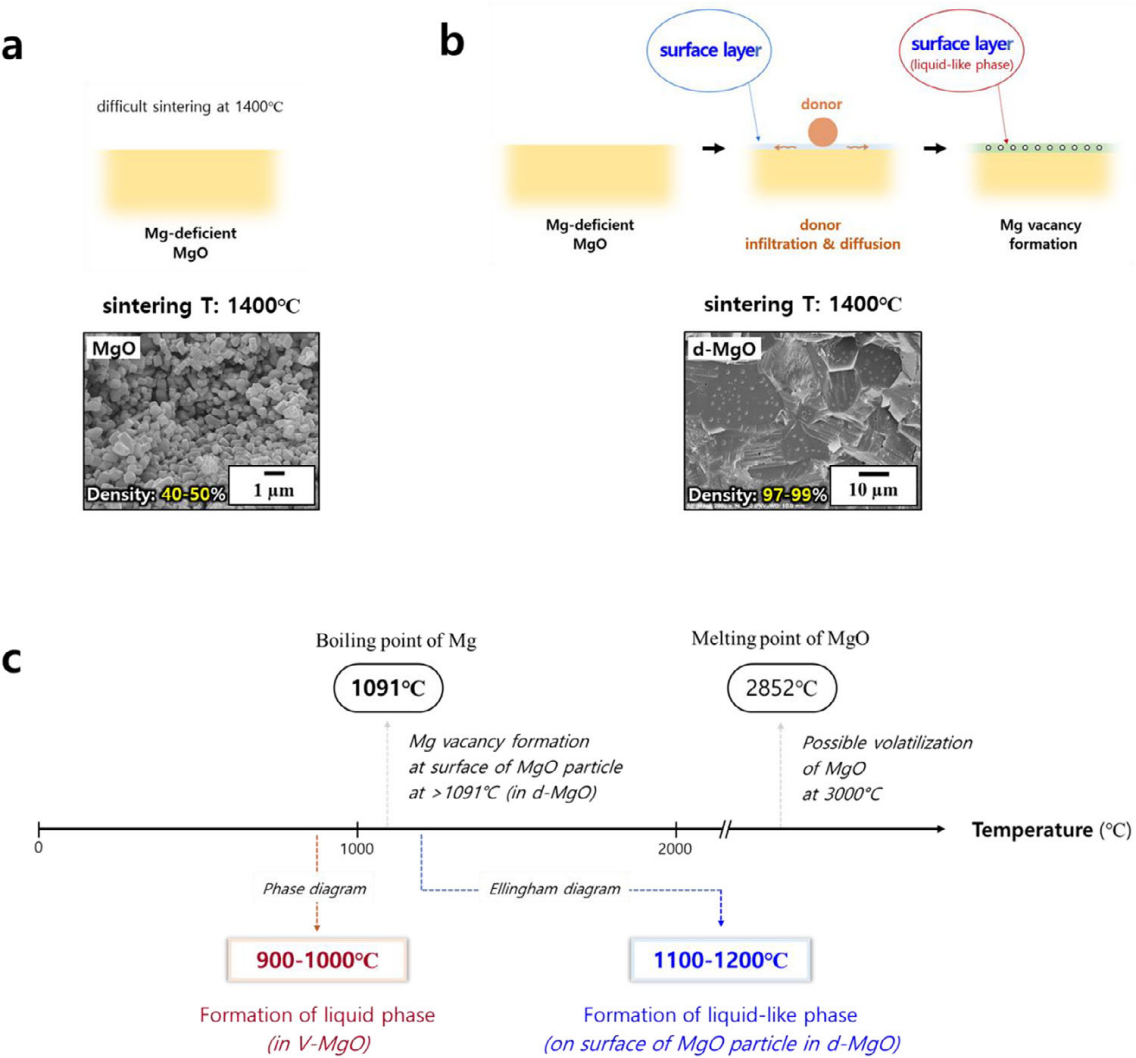
Distinct sintering behavior in undoped MgO, d‐MgO, and V‐MgO. a) Difficult sintering of MgO at 1400 °C and b) liquid phase sintering of d‐MgO. c) Low‐temperature sintering distinction: V‐MgO via phase diagram insights, d‐MgO via Ellingham diagram principles.

As demonstrated in Figure S8 (Supporting Information), MgO is generally understood to be magnesium‐deficient. As shown in **Figure** **3**a, sintering at 1400 °C results in poor grain growth and densification. In contrast, Figure 3b reveals that d‐MgO exhibits liquid‐phase sintering behavior, driven by the formation of numerous Mg vacancies at the particle surfaces due to donor doping. These vacancies promote liquid‐like behavior at the surfaces of MgO particles, which in turn facilitates grain growth and densification, as clearly evidenced by the SEM image.

The donor concentration (Ti^4+^: 0.15 at.%, Nb^5+^: 0.10 at.%) employed in this study may be perceived as insufficient to drive the formation of a liquid phase. Therefore, it is important to note that the donor does not need to induce Mg vacancies throughout the entire particle. Instead, it only needs to generate localized vacancies at the particle surfaces to initiate liquid‐phase formation. Therefore, only a minimal amount of donor is required. In fact, excessive donor addition leads to adverse effects, such as the formation of secondary phases and suppression of grain growth, as confirmed in Figure S9 (Supporting Information).

V‐MgO, which utilizes liquid phase formation driven by the eutectic point identified in the phase diagram (Figure S1, supporting information), can be sintered at a lower temperature compared to TN‐MgO. Figure 3c summarizes the distinct differences of sintering behaviors in V‐MgO and d‐MgO, as inferred from the phase diagram (Figure S1, Supporting Information), Ellingham diagram,^[^ ^51^, ^52^ ^]^ and DTA results (Figure S7, Supporting Information).

As shown in Figure S11 (Supporting Information), the sintering behavior of MgO can also be adequately explained by conventional sintering theory. The non‐linearity suggests the co‐existence of two distinct kinetics of grain boundary migration, depending upon the driving force for migration (Δ*g*), as expressed by the following equation:^[^ ^54^ ^]^

(3) $$\Delta g=4\gamma_{b}\left(1/\bar{G}-1/G\right)$$

where $\bar{G}$ is the mean grain size, *G* is the size of each grain and γ_*b*_ is the grain boundary energy. This equation describes the relationship between mean grain size in a polycrystalline system and the size of individual grains, providing a range of driving force for grain growth as shown by the bar in Figure S11 (Supporting Information). In general, this mechanism is considered to understand the sintering behavior of a ceramic material, but it is not an easy approach for the readers. Therefore, in this study, we described the sintering behavior with the variation of the particle surface, as indicated in Figure 3.

### 2.3 High Thermal Conductivity of MgO

**Figure 4:**
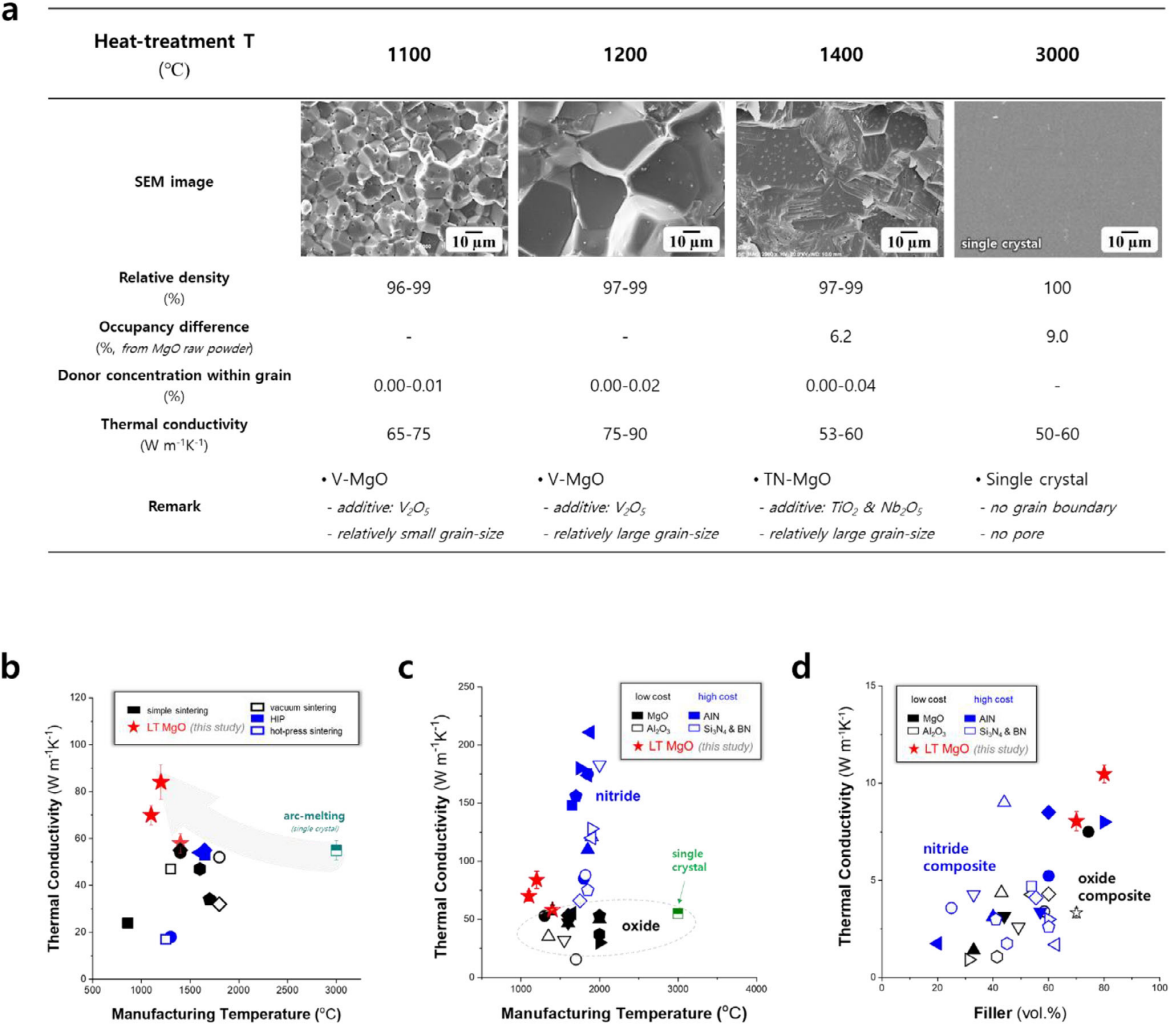
Thermal conductivity values and microstructural attributes of MgO ceramics prepared across a spectrum of processing temperatures. a,b) Thermal conductivities of MgO‐based materials capable of exhibiting high thermal performance at temperatures below the melting point of MgO (2852 °C).^[^ ^30^, ^31^, ^39^, ^40^, ^41^, ^42^, ^43^, ^44^, ^45^, ^46^, ^47^, ^48^ ^]^ c) Benchmarking of thermal conductivities exhibited by diverse heat‐dissipative ceramic systems,^[^ ^30^, ^31^, ^34^, ^39^, ^41^, ^49^, ^50^, ^51^, ^52^, ^53^, ^54^, ^55^, ^56^, ^57^, ^58^, ^59^, ^60^, ^61^, ^62^, ^63^, ^64^, ^65^, ^66^, ^67^, ^68^, ^69^, ^70^, ^71^, ^72^, ^73^, ^74^, ^75^, ^76^ ^]^ highlighting superior performance of MgO (> 80 W m^−1^ K^−1^). d) Comparative assessment of thermal conductivities in various TIMs,^[^ ^31^, ^77^, ^78^, ^79^, ^80^, ^81^, ^82^, ^83^, ^84^, ^85^, ^86^, ^87^, ^88^, ^89^, ^90^, ^91^, ^92^, ^93^, ^94^, ^95^, ^96^, ^97^, ^98^, ^99^, ^100^, ^101^, ^102^ ^]^ demonstrating competitive edge of MgO‐based TIMs over nitride‐based counterparts. The occupancy difference and donor concentration within the grains is presented in detail in Figures S8 and S12 (Supporting Information). The occupancy difference obtained from Rietveld refinement does not carry absolute quantitative significance, but rather represents a relative comparison among specimens.

**Figure** **4**a,b presents the relative densities, occupancy difference, donor concentration within grain, thermal conductivities, and corresponding SEM images of MgO specimens prepared at various temperatures.^[^ ^30^, ^31^, ^39^, ^40^, ^41^, ^42^, ^43^, ^44^, ^45^, ^46^, ^47^, ^48^ ^]^ All specimens exhibited high relative densities (≥ 96%), with the MgO single‐crystal exhibiting 100%. As shown in the SEM image in Figure 4a, no pores were observed in the single‐crystal.

However, given that the fabrication temperature of single‐crystal MgO is near 3000 °C and involves melting the MgO material, the possibility of MgO volatilization during processing cannot be ruled out, and the resulting formation of magnesium vacancies cannot be neglected. Accordingly, as shown in the Rietveld refinement results presented in Figure S8 (Supporting Information), the Mg occupancy is lowest in the single‐crystal MgO, followed by d‐MgO sintered at 1400 °C, with the raw MgO powder exhibiting the highest occupancy. This trend suggests that the concentration of Mg vacancies can be highest in the single‐crystal and comparatively lower in the raw powder. The notably low Mg occupancy observed in the single‐crystal is likely attributable to MgO volatilization during high‐temperature processing, a possibility that cannot be overlooked.

As shown in Figure 4a, TN‐MgO exhibits a higher thermal conductivity than single‐crystalline MgO, despite the presence of grain boundaries and internal pores. A previous study has demonstrated that, in MgO ceramics, once the grain size and relative density reach sufficiently high levels, point defects emerge as the dominant phonon‐scattering centers limiting thermal conductivity.^[^ ^31^ ^]^ Remarkably, as revealed in Figure S12 (Supporting Information), Ti and Nb were partially incorporated into the grain interiors of MgO, yet the material still demonstrates superior thermal transport performance. This observation can be attributed to the fact that the incorporated concentrations of Ti and Nb may be exceedingly low, and the resulting Mg vacancy concentration is still lower than that of single‐crystalline MgO, where substantial magnesium volatilization likely occurred during fabrication.

Accordingly, as demonstrated in Figures S13–S17 (Supporting Information), the impact of crystallographic point defects, including magnesium vacancies, on the thermal conductivity of MgO was quantitatively assessed through first‐principles calculations. Comprehensive results of the computational assessment are presented in Figures S13 and S14 (Supporting Information). As a result, Figure S14 (Supporting Information) indicates that magnesium or oxygen vacancies exert a more significant detrimental effect on the thermal conductivity of MgO compared to other types of defects. In this study, magnesium deficiency was experimentally confirmed to be more pronounced than oxygen deficiency; therefore, magnesium vacancies were primarily considered and comparatively analyzed.

Experimentally, the trend in magnesium vacancy concentration suggests that the as‐received MgO powder contains a substantially lower concentration of magnesium vacancies than single‐crystalline MgO, as evidenced in Figure S8 (Supporting Information). In the case of the V‐MgO specimen, vanadium is partially incorporated into the grain interiors, which may introduce some level of magnesium vacancies and contribute to a reduction in thermal conductivity.

Nevertheless, SEM micrographs presented in Figure S4d (Supporting Information) and Figure 4a indicate that V‐MgO possesses more pronounced liquid‐phase features along the grain boundaries in comparison to TN‐MgO. The liquid phase is composed of Mg, V, and O, indicating that a substantial portion of the vanadium is concentrated within this intergranular phase. In addition, compositional data from Figure S12 (Supporting Information) suggest that the amount of donor species incorporated into the grain interiors is lower in V‐MgO than in TN‐MgO, leading to a relatively lower concentration of magnesium vacancies. Based on these findings, V‐MgO can exhibit higher thermal conductivity than TN‐MgO due to its reduced point‐defect concentration. This observation underscores the critical role of point defects, even at trace concentrations, in limiting thermal transport.^[^ ^35^, ^36^, ^37^, ^38^ ^]^

In V‐MgO and TN‐MgO, the extent of Mg vacancy formation may indirectly evaluated by analyzing the degree of donor incorporation into the interior of MgO grains via cation substitution. The substitutional incorporation ratios of Ti, Nb, and V within the MgO grains were quantified through TEM‐based compositional analysis, as shown in Figure 4a and Figure S12 (Supporting Information). While donor elements were abundantly present in the liquid phase at grain boundaries and surfaces, only a trace amount (0.00–0.04 at.%) was substitutionally incorporated into the grain interiors. Notably, V‐MgO exhibited a slightly lower level of donor incorporation into the MgO grains compared to TN‐MgO. Considering the limitations of TEM‐EDS analysis, the absolute quantification of donor concentrations is likely overestimated, and only relative comparisons among samples should be regarded as meaningful. As illustrated in Figures S13 and S14 (Supporting Information), the high thermal conductivity presented in Figure 4a is feasible, and the point defect concentrations in V‐MgO and TN‐MgO are expected to be extremely low, likely below 0.01%.

To quantitatively assess the tendency of thermal conductivities, the relationship between Mg vacancy concentration and thermal conductivity was theoretically modeled and calculated in this study (Figures S13–S17, Supporting Information). Based on the calculations presented in Figure S14 (Supporting Information), the Mg vacancy concentration was estimated to fall within the range of 0.01–0.1 at.% in single‐crystalline MgO.

These results suggest that the Mg vacancy concentration, which is modulated by sintering behavior and additives, is a dominant factor governing thermal transport in MgO. Nevertheless, grain size also plays a critical role. For example, V‐MgO sintered at 1100 °C exhibits lower thermal conductivity than that sintered at 1200 °C due to its smaller grain size.

As a result, the TC of MgO was significantly improved under the constrained processing conditions, it remains lower than that of typical nitrides (Figure 4b),^[^ ^30^, ^31^, ^34^, ^39^, ^41^, ^49^, ^50^, ^51^, ^52^, ^53^, ^54^, ^55^, ^56^, ^57^, ^58^, ^59^, ^60^, ^61^, ^62^, ^63^, ^64^, ^65^, ^66^, ^67^, ^68^, ^69^, ^70^, ^71^, ^72^, ^73^, ^74^, ^75^, ^76^ ^]^ which are known for their superior heat dissipation properties. Nitrides, however, are costly due to their high process temperatures (1800–2000 °C) and the requirement for non‐oxidizing atmospheres. Despite this, the difference in TC between nitride‐based TIMs and LT MgO TIMs is minimal (Figure 4d), due to the excellent surface properties of LT MgO fillers.^[^ ^31^, ^77^, ^78^, ^79^, ^80^, ^81^, ^82^, ^83^, ^84^, ^85^, ^86^, ^87^, ^88^, ^89^, ^90^, ^91^, ^92^, ^93^, ^94^, ^95^, ^96^, ^97^, ^98^, ^99^, ^100^, ^101^, ^102^ ^]^

### 2.4 Excellent Cooling Performance of LT MgO TIM for BP

**Figure 5:**
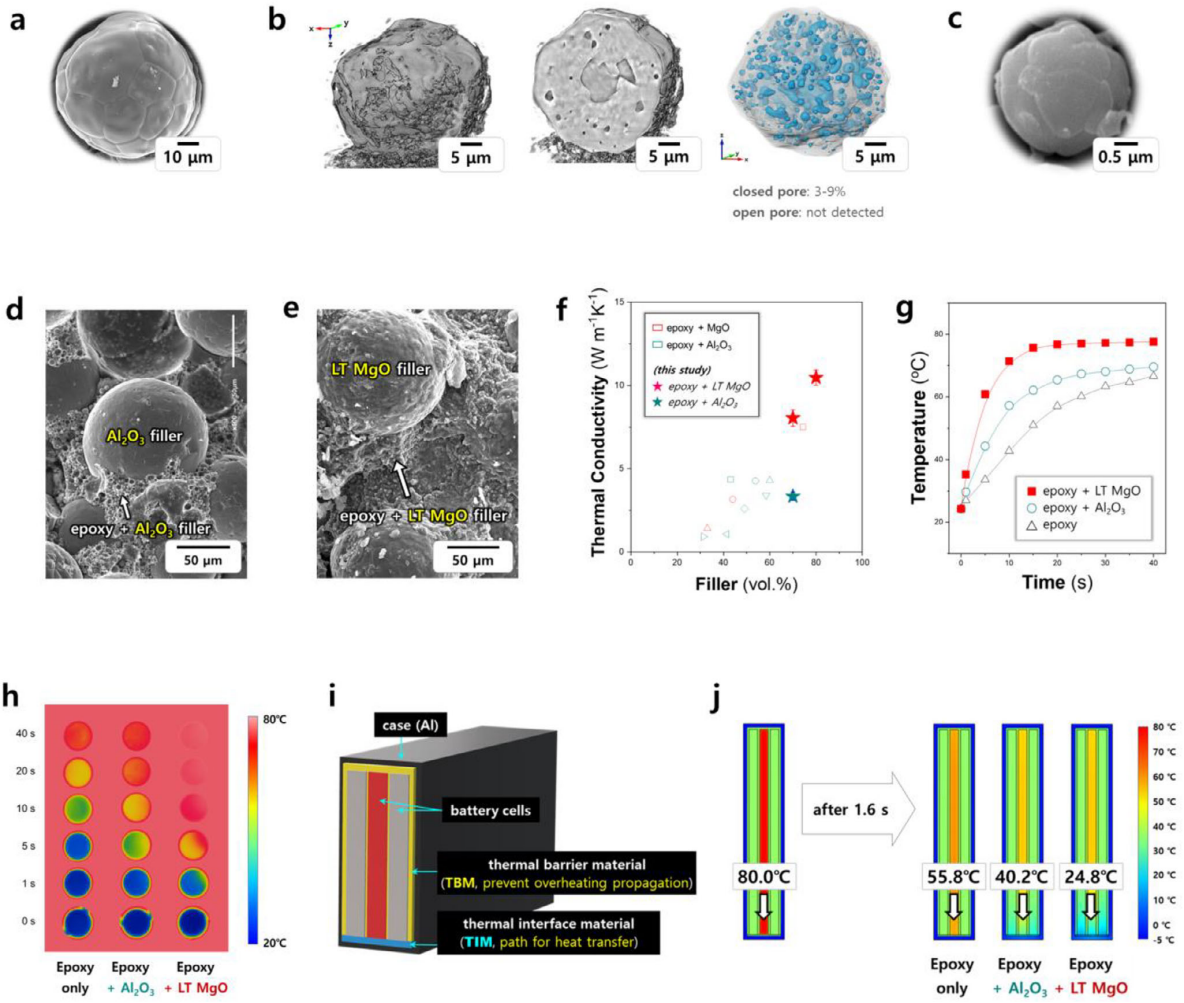
LT MgO fillers, TIMs, and heat dissipation performance. a–c) Microstructures of LT MgO fillers. d,e) SEM images of Al_2_O_3_ TIM and LT MgO TIM. f) Thermal conductivities of epoxy composites incorporating various oxide fillers.^[^ ^31^, ^77^, ^78^, ^79^, ^80^, ^81^, ^82^, ^83^, ^84^, ^85^ ^]^ g,h) Temperature profiles of neat epoxy and epoxy‐oxide filler composites over heating time (bottom temperature: 80 °C). i) Simplified thermal interface boundary layer (TIBM) model of a battery module. j) Temperature variations within an 80 °C cell after 1.6 s, demonstrating the effect of TIMs with thermal conductivities of 0.2, 3.0, and 10.0 W m^−1^ K^−1^. Microstructural analyses were performed using SEM and nano‐CT (X‐ray Zernike phase‐contrast nano‐tomography measurements, Pohang Accelerator Laboratory) imaging. Notably, no open pores were observed in the MgO filler, and the cooling performance of the LT MgO TIM significantly surpassed that of a commercial Al_2_O_3_ TIM. Heat transfer within the battery package was modeled using COMSOL Multiphysics 5.3a (Figures S21–S27, Supporting Information).

To prepare TIMs for the thermal management of BP, LT MgO fillers were well dispersed within a polymer matrix. Spherical fillers generally promote superior mixing with polymers, and smoother surface morphologies typically enhance this compatibility. Consequently, spherical LT MgO fillers of various sizes were synthesized, exhibiting excellent surface properties, as detailed in **Figures** **5**a–c and S18 (Supporting Information). For comparative analysis, commercial Al_2_​O_3_​ fillers, also characterized by smooth surfaces (Figure 5d), were included. Notably, the LT MgO fillers displayed no detectable open porosity, suggesting optimal dispersion within the epoxy polymer used in this study.

Indeed, both LT MgO and Al_2_O_3_ TIMs were successfully fabricated, as evidenced by Figure 5d,e and Figure S19 (Supporting Information). The resulting LT MgO TIMs achieved high thermal conductivities ranging from 8.0 to 10.0 W m^−1^K^−1^, as illustrated in Figure 5f. Furthermore, the high thermal conductivity of the LT MgO TIMs led to significantly accelerated heat transfer compared to commercial Al_2_O_3_ TIMs, as demonstrated in Figure 5g,h and Figure S20 (Supporting Information).

As illustrated in Figure 5i, the thermal barrier materials (TBMs) are also employed in BPs in addition to TIMs. The model of TIM and TBM (TIBM) model, depicted in Figure 5i, differs from the conventional TIM model (Figure 1a) and is increasingly adopted in electric vehicles. Its primary function is to prevent the propagation of high temperatures from rapidly heated cells to adjacent cells, thereby enhancing safety.

Similar to the TIM model, we simulated the scenario where the center cell's temperature abruptly rises to 80 °C to evaluate the cooling performance of commercial Al_2_O_3_ TIM versus LT MgO TIM within the TIBM configuration. As shown in Figure 5j, the cell incorporating the 10.0 W m^−1^K^−1^ TIM (presumably LT MgO TIM) cooled more effectively to 24.8 °C, while the commercial cell with a 3.0 W m^−1^K^−1^ TIM (commercial Al_2_O_3_ TIM) only cooled to 40.2 °C, both after 1.6 s. Further simulation results, encompassing various conditions such as air temperature, battery cell temperature, and BP structure (all of which influence battery cell cooling rates), are summarized in Figures S21–S27 (Supporting Information).

Consequently, our findings clearly demonstrate that replacing commercial Al_2_O_3_ TIM with LT MgO TIM significantly enhances the cooling performance of the battery pack and, by extension, the safety of electric vehicles.

## 3 Conclusion

This study demonstrates that MgO can achieve a TC comparable to that of high‐performance nitride materials, while offering superior cost‐effectiveness and processability. The TC of MgO is primarily governed by the concentration of magnesium vacancies, which was shown to be tunable through the precise selection of minimal dopant concentrations and the careful optimization of processing parameters. Moreover, donor doping promotes the LP formation on the surfaces of MgO particles, thereby enabling densification at reduced temperatures. As a result, d‐MgO can be sintered at 1100–1400 °C, which is significantly lower than the typical sintering temperature of commercial alumina (1500–1600 °C).

Given these findings, MgO emerges as a promising candidate to replace conventional alumina in TIM applications for battery packaging, offering a scalable and thermally robust solution to enhance the safety of electric vehicles. As demonstrated in this study, the integration of LT‐MgO TIMs leads to a threefold improvement in battery cell cooling rates compared to commercial TIM counterparts.

## 4 Experimental Section

MgO (99.9%, High‐Purity Chemicals, Japan), TiO_2_, Nb_2_O_5_, B_2_O_3_, and V_2_O_5_ (99.9%, all obtained from Sigma–Aldrich, USA) powders were mixed by ball‐milling to prepare a TN‐MgO (MgO + 0.15 at.% TiO_2_ + 0.1 at.% Nb_2_O_5_), B‐MgO (MgO + 0.3 at.% B_2_O_3_), and V‐MgO (MgO + 0.3 at.% V_2_O_5_). They were dried and pressed into disks by cold isostatic pressing at 200 MPa. The disks were then sintered at 1100–1400 °C. MgO materials were also sintered at 1100–1400 °C for 2 h for comparison. The heating rate was 5 °C min^−1^, whereas the cooling rate was not controlled (furnace cooling). The MgO single crystals were prepared from a product manufactured via a specialized arc‐melting process and purchased from MTI Corp. (≥ 99.95%, USA).

In order to prepare a MgO filler, MgO and additives were mixed with a binder composed of polyvinyl butyral (B‐76, Eastman) and dioctyl phthalate (Sigma–Aldrich) for 24 h by ball‐milling to prepare a slurry for spray‐drying. Granulation by spray‐drying was performed using a spray dryer (DJE‐FCNM‐022R; Dongjin Eng. Korea) to prepare spherical MgO particles as fillers, which were subsequently heat‐treated at 1100–1400 °C for 2 h in an electric furnace (Elevator Furnace, TNS Vacuum, Korea). The heating rate was 5 °C min^−1^, whereas the cooling rate was not controlled (furnace cooling). The MgO fillers were classified by size (20, 60, and 100 µm) using an automatic sieve and evaluated using a prototype that was mass‐produced by a ceramics company (Soulmaterial, Korea). The Al_2_O_3_ fillers (DAW‐90, DAW‐20, and DAW‐3) were purchased from Denka Co., Ltd.

The small MgO fillers with an average particle size of ≈1 µm were synthesized using a polymeric precursor method followed by spray drying and subsequent heat treatment. The precursor solution was prepared by dissolving the following components in distilled water under continuous magnetic stirring at room‐temperature: 0.1 m magnesium nitrate hexahydrate (Mg(NO_3_)_2_·6H_2_O, Junsei, Japan) as the magnesium source, 0.0003 m ammonium metavanadate (NH_4_VO_3_, Sigma–Aldrich, USA) as a dopant, 0.1 m citric acid monohydrate (C_6_H_8_O_7_·H_2_O, Samchun, Korea), and 0.1 m ethylene glycol (C_2_H_6_O_2_, Samchun, Korea) as a polymerization agent. The solution was stirred until a clear and homogeneous mixture was obtained. The resulting polymeric precursor solution was subjected to a spray‐drying process using a laboratory‐scale spray dryer. The atomization was carried out through a two‐fluid nozzle system operated at an atomizing pressure of 2.0 bar. The inlet and outlet temperatures of the spray dryer were maintained at 200 °C and 110 °C, respectively, to facilitate rapid solvent evaporation and particle formation. The as‐dried powders were then subjected to a two‐step thermal treatment in a furnace under ambient air atmosphere to induce organic decomposition and crystalline phase formation. The first calcination step was performed at 900 °C for 2 h to remove residual organics and initiate crystallization. Subsequently, a second heat treatment was conducted at 1100 °C for 2 h to promote grain growth and densification of the MgO particles. The heating and cooling rates for both steps were set to 5 °C min^−1^ to ensure thermal uniformity.

TIMs were prepared from MgO fillers (ExAl100, ExAl60, ExAl20, and a small MgO filler, Korea Institute of Materials Science and Soulmaterial) and Al_2_O_3_ fillers using a simple mixing process. An epoxy solution was used as polymer for these composites. The epoxy solution was prepared from the diglycidyl ether of bisphenol‐A (YD‐128, Kukdo Chemical, Korea), methyl tetrahydrophthalic anhydride (Kukdo Chemical, Korea), and *N*,*N*‐dimethylbenzylamine (Sigma–Aldrich) at a weight ratio of 100:90:0.5. The MgO fillers (100 and 1 µm) and Al_2_O_3_ fillers (90, 20, and 3 µm) were mixed in weight ratios of 8:2 and 20:7:7 ratio, respectively, and incorporated into the epoxy resin volume fraction of 80 and 70 vol.%. After mixing, the slurry was placed in a stainless‐steel mold (diameter: 12.7 mm), deformed, and precured in a vacuum desiccator at 150 °C for 20 min. Thereafter, the precured mixture in the mold was cured at 150 °C for 2 h in a drying oven. The thickness of the specimen was controlled to be 1.0 mm to measure the thermal conductivity.

### Analyses of Microstructure and Crystal Structure

The crystal structures of the specimens were analyzed using X‐ray diffraction (XRD). High‐resolution synchrotron powder XRD data were obtained using the 9B high‐resolution powder diffractometer (HRPD) beamline of Pohang Light Source II (PLS‐II) in Korea. The XRD data were collected in the 2θ range of 30°–110° with a step size of 0.005° at an incident wavelength of 1.5213 Å. Rietveld refinement of the recorded HRPD patterns was performed using Fullprof to obtain the structural parameters of the samples.

The microstructures and crystal structure of the samples were additionally observed using a scanning electron microscope (SEM, JSM‐6610LV; JEOL Ltd., Japan), electron probe microanalysis (EPMA, JEOL/JXA‐8530F), and transmission electron microscope (TEM, JEM‐2100F, JEOL Ltd., Japan). X‐ray Zernike phase‐contrast nanotomography measurements were performed using the 7C XNI nanoimaging beamline of PLS‐II in Korea, which is designed for both spectral microscopy and nanotomography by utilizing zone‐plate‐based full‐field transmission X‐ray microscopy. Monochromatic X‐rays (9.3 keV) were selected using a liquid‐nitrogen‐cooled double‐crystal monochromator and focused using rhodium‐coated horizontal and vertical focusing plane mirrors. A gold objective zone plate with an outermost zone width of 30 nm, diameter of 300 µm, and thickness of 0.9 µm was used for nanotomography. The Zernike phase‐contrast method was applied using a holed gold‐film phase plate with a thickness of 960 nm to obtain a high‐contrast image. A scintillator‐coupled optical microscope incorporating a thin scintillation crystal (20‐µm‐thick GAGG:Ce), 20× optical objective, and high‐end scientific complementary metal–oxide–semiconductor camera with 2048 × 2048 sensors and a pixel size of 6.5 µm was used as the detector. In this experiment, the field of view was 50 µm, and the effective pixel size (with two bins) was equivalent to 50 nm. For each tomography scan, 1000 projections were acquired with an exposure time of 1 s. Tomographic reconstruction was conducted using the filtered back‐projection algorithm method (Octopus software). Segmentation and visualization of the 3D data were performed using the Avizo software package.

### Property Measurement

Thermal conductivity (*K*) was calculated using the relation *K* = *ρC_p_α* (laser‐flash method, ASTM E1461‐13). Thermal diffusivity (*α*) was measured using the laser flash technique (LFA 467 Hyperflash, NETZSCH, Germany). Specific heat (*C_p_*) was evaluated using a differential scanning calorimeter (Q2000, TA Instruments, USA) with a sapphire standard. The densities (*ρ*) of the specimens were measured using Archimedes’ method with *m*‐xylene as the medium. All properties were measured at 25 °C. The heat transport properties of the specimens were characterized using an infrared thermal imaging camera (IR camera, Xi 400 cam, Optris, Germany) with an IR resolution of 382 × 288 pixels. All specimens were simultaneously placed on an aluminum plate and heated from 30 to 100 °C, after which their temperature variation over time was observed using the IR camera.

### Heat Transfer Simulation of Battery Package

The simulation was conducted using COMSOL Multiphysics 5.3 to evaluate the cooling behavior of the battery cell as a function of the thermal conductivity of TIM materials. Schematic diagrams of the models are provided in Figure S21 (Supporting Information), and the structural dimensions and thermophysical properties are summarized in Table S5 (Supporting Information).

The initial temperature of the central battery cell was set to 80 °C, and the surrounding materials were set to initial temperatures between 20 and 40 °C. The thermal conductivity of battery cells varies widely as a function of structure and material composition. Accordingly, a broad range of values (0.25–200 W m^−1^ K^−1^) was considered in this study. (Figures S22–S24, Supporting Information).

Only heat conduction was considered in the simulation, excluding electrochemical reactions and internal heat sources. The Heat Transfer in Solids module in COMSOL Multiphysics was used to solve the time‐dependent heat conduction equation:

(4) $$\rho \mathrm{Cp}\frac{\Delta T}{\Delta t}=\nabla \left(k\nabla T\right)+Q$$

where *ρ* is the density, *C_p_* is the specific heat capacity, *k* is the thermal conductivity, and *Q* is the external heat source (set to zero in this study). In this simulation, natural convection between the battery surface and ambient air was applied as the surface boundary condition. The heat flux (*q_0_*) at the boundary was defined as:

(5) $$q_{0}=h\left(T_{\mathrm{ext}}-T\right)$$

where ℎ is the heat transfer coefficient (set to 10 W m^−2^ K^−1^ to represent typical natural convection in air), *T_ext_* is the external (ambient) temperature, and *T* is the battery module surface temperature. The external temperature ranged from −5 to 40 °C to account for varying environmental conditions. (Figures S25 and S26, Supporting Information) A fine mesh was applied to ensure the accuracy and stability of the simulation results.

### Thermogravimetry (TG)/Differential Thermal Analysis (DTA)

TG/DTA (STA 409 PC, NETZSCH, Germany) was performed to determine the temperature at which the liquid‐like phase forms in TN‐MgO and the temperature at which Mg volatilizes in magnesia. The temperature increase rate was 5 °C min^−1^, and heating was performed from 20 to 1400 °C.

### Computational Methods

First‐principles calculations were performed using the Vienna Ab initio simulation package (VASP) with the projector‐augmented‐wave (PAW) method and the local density approximation (LDA) for the exchange‐correlation functional.^[^ ^103^, ^104^, ^105^ ^]^ A plane‐wave energy cutoff of 520 eV was used. The primitive cell was fully relaxed with a total energy convergence threshold of 10^−8^ eV and a force convergence threshold of 10^−7^ eV Å^−1^, using a 24 × 24 × 24 **k**‐mesh. The second‐ and third‐order interatomic force constants were computed using Phonopy and ThirdOrder, respectively. A 4 × 4 × 4 supercell (128 atoms) with a 6 × 6 × 6 **k**‐mesh was used, and an energy convergence threshold of 10^−8^ eV was applied.^[^ ^106^, ^107^ ^]^ The calculated phonon dispersion relations agree with experimental data well, better than predicted by using PBE exchange‐correlation functional in the literature (Figure S16, supporting information).^[^ ^108^ ^]^ The third‐order force constants included interactions up to the sixth nearest neighbors, with atomic displacements of 0.03 Å. Non‐analytical corrections (NAC) were applied to account for longitudinal optical–transverse optical (LO–TO) phonon splitting at the Γ point in phonon dispersion calculations.^[^ ^109^, ^110^ ^]^ Phonon scattering rates and thermal conductivity were calculated by solving BTE using a fully iterative approach by ShengBTE. Convergence tests were carried out with respect to **q**‐mesh and Gaussian smearing width (Figure S13, supporting information).^[^ ^111^ ^]^ Phonon‐point defect scattering was calculated using the Tamura formalism, extended by Klemens to incorporate the effect of local bonding perturbations.^[^ ^112^, ^113^, ^114^ ^]^ The scattering rates are shown in Figure S17 (supporting information). Phonon renormalization and four‐phonon scattering are not considered since they play marginal roles at room temperature and tend to cancel out each other. Their impact would be buried by the impact of choice of exchange correlation functionals.

## Conflict of Interest

The authors declare no conflict of interest.

## Supporting information



Supporting Information

## Data Availability

The data that support the findings of this study are available from the corresponding author upon reasonable request.
