# Supplementary material for: Advanced Thermal Interface Materials: Insights into Low‐Temperature Sintering and High Thermal Conductivity of MgO
Source: Adv Mater. 2025 Aug 27;37(45):e10237. doi: 10.1002/adma.202510237 (PMC12617043; doi:10.1002/adma.202510237)
Supplement: Supplementary file 1 — Supporting Information [file ADMA-37-e10237-s001.pdf]

ADVANCED MATERIALS

Supporting Information

for *Adv. Mater.*, DOI 10.1002/adma.202510237

Advanced Thermal Interface Materials: Insights into Low-Temperature Sintering and High Thermal Conductivity of MgO

Su-Jin Ha, Hye-Jeong Jang, Hui-jin Son, Young Kook Moon, Hyun-Ae Cha, Jong-Jin Choi, Jee-Hyuk Ahn, Byung-Dong Hahn, Kyung-Hoon Cho*, Do-Cheon Ahn, Jun Lim, Sang-Chae Jeon, In Chul Jung, Youngsup Song, Hao Zhou, Tianli Feng and Cheol-Woo Ahn**

Supporting Information

Advanced Thermal Interface Materials: Insights into Low-Temperature Sintering and High Thermal Conductivity of MgO

Su-Jin Ha, Hye-Jeong Jang, Hui-jin Son, Young Kook Moon, Hyun-Ae Cha, Jong-Jin Choi, Jee-Hyuk Ahn, Byung-Dong Hahn, Kyung-Hoon Cho*, Do-Cheon Ahn, Jun Lim, Sang-Chae Jeon, In Chul Jung, Youngsup Song, Hao Zhou, Tianli Feng, Cheol-Woo Ahn**

Corresponding author: cera72@kims.re.kr, cheoruahn@kims.re.kr

Table of Contents

Figs. S1 to S26 (2 p to 30 p)

Tables S1 to S5 (31 p to 35 p)

References (36 p)

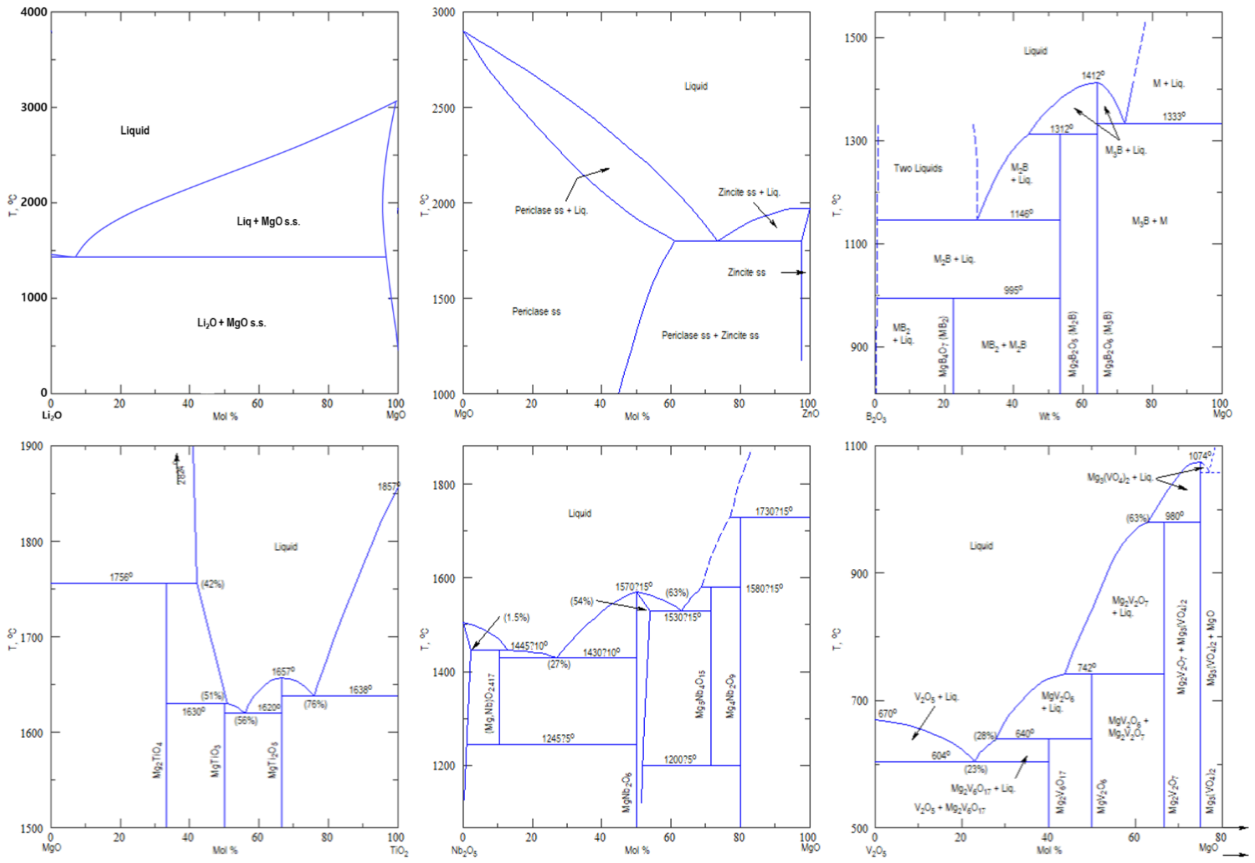

Figure S1. Phase diagrams of MgO-Li_2O/ZnO/B_2O_3/TiO_2/Nb_2O_5/V_2O_5.[1-6]

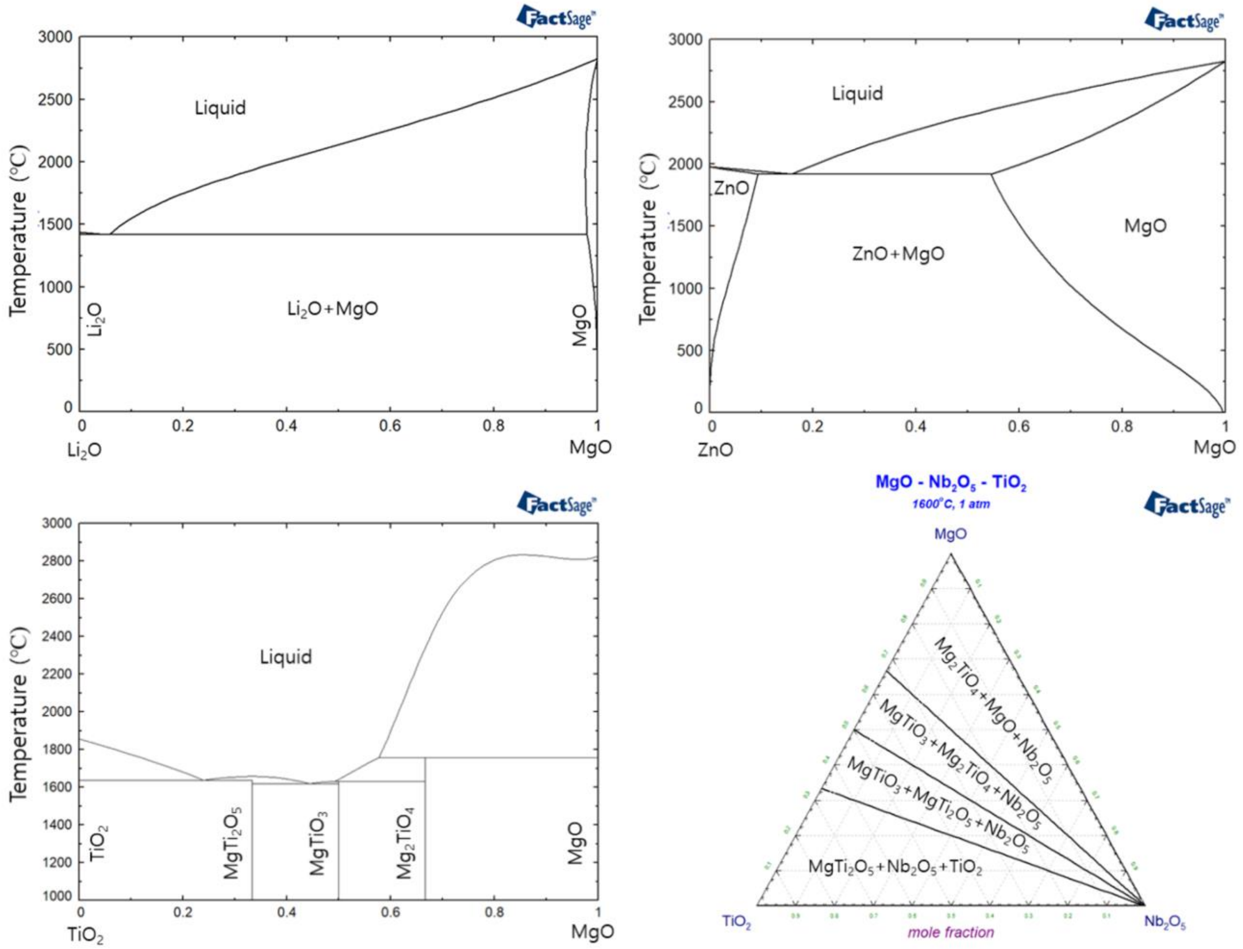

Figure S2. Phase diagrams of MgO-Li_2O/ZnO/TiO_2/Nb_2O_5 plotted using FactSage 8.1 (Thermfact/CRCT, Montreal, QC, Canada) thermodynamic software with database of FactPS, FToxid and FTlite.

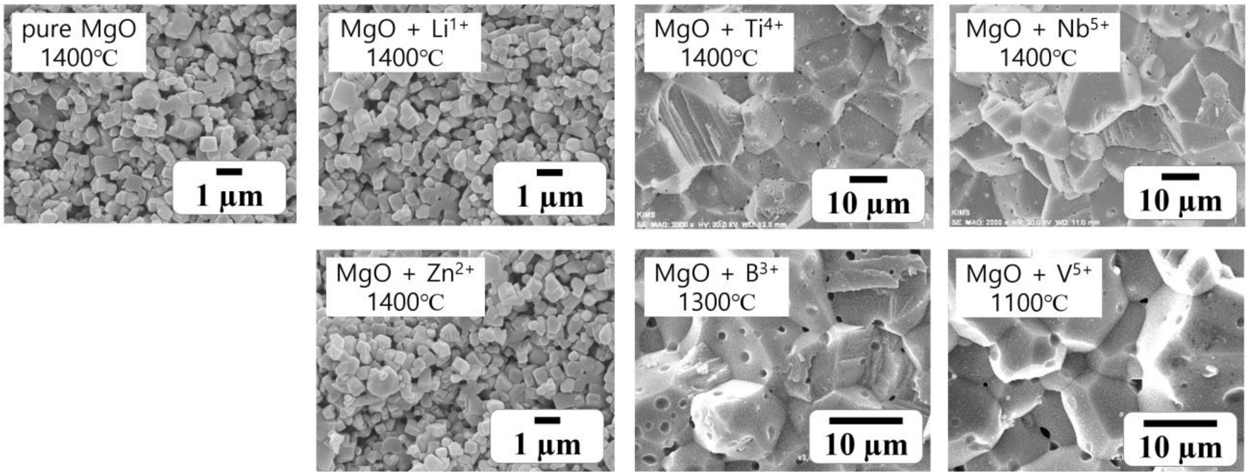

Figure S3. SEM images of MgO materials sintered at 1100-1400 ℃.

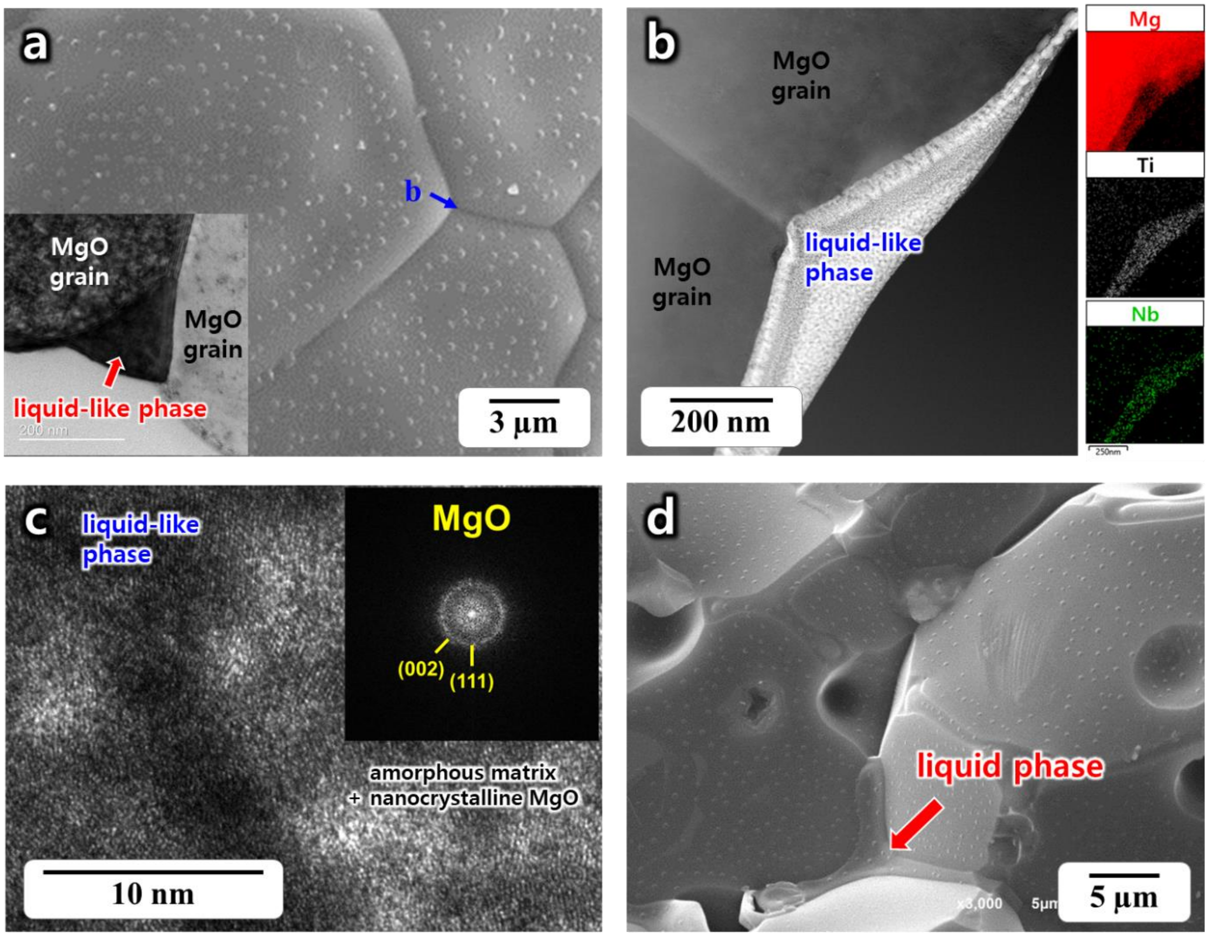

Figure S4. SEM and TEM images of (a-c) TN-MgO and (d) V-MgO. The liquid-like phase is found in the grain boundaries. Liquid phase is observed at the grain boundaries of V-MgO.

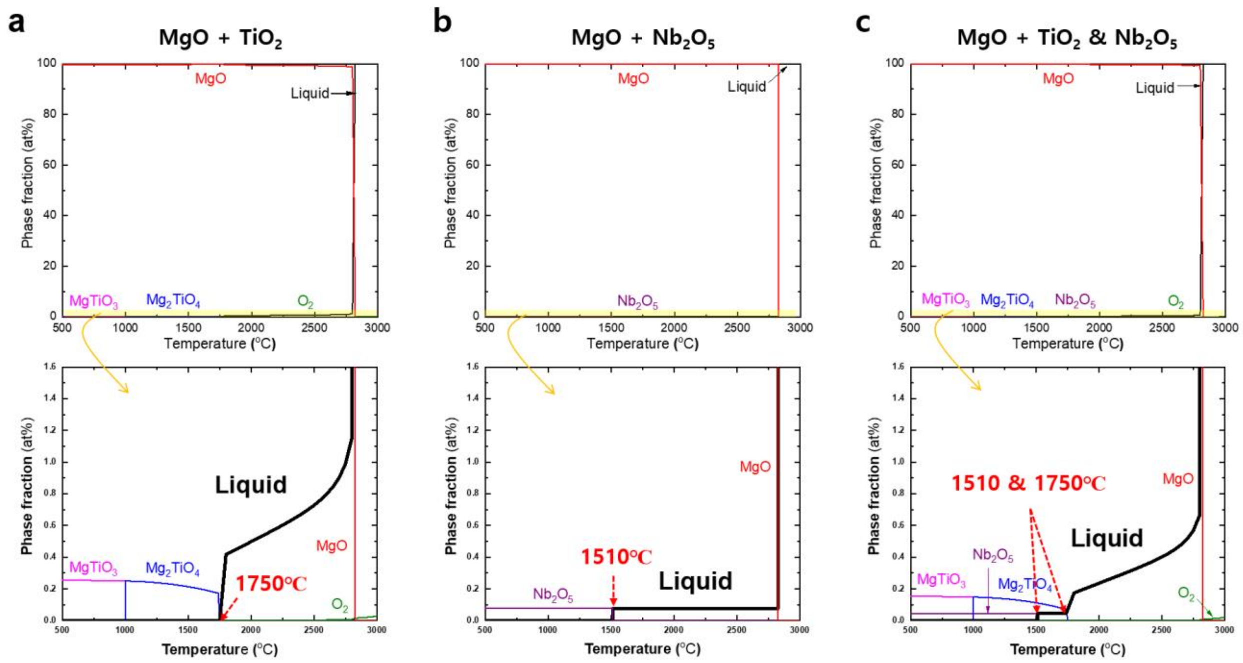

Figure S5. Phase diagrams of (a) MgO + Ti^{4+}, (b) MgO + Nb^{5+}, and (c) MgO + Ti^{4+} & Nb^{5+} (plotted using FactSage 8.1, Thermfact/CRCT, Montreal, QC, Canada) thermodynamic software with database of FactPS, FToxid and FTlite. According to phase diagrams, LP in MgO does not form at the temperatures below 1,400 °C, when Ti^{4+} or Nb^{5+} is added to MgO.

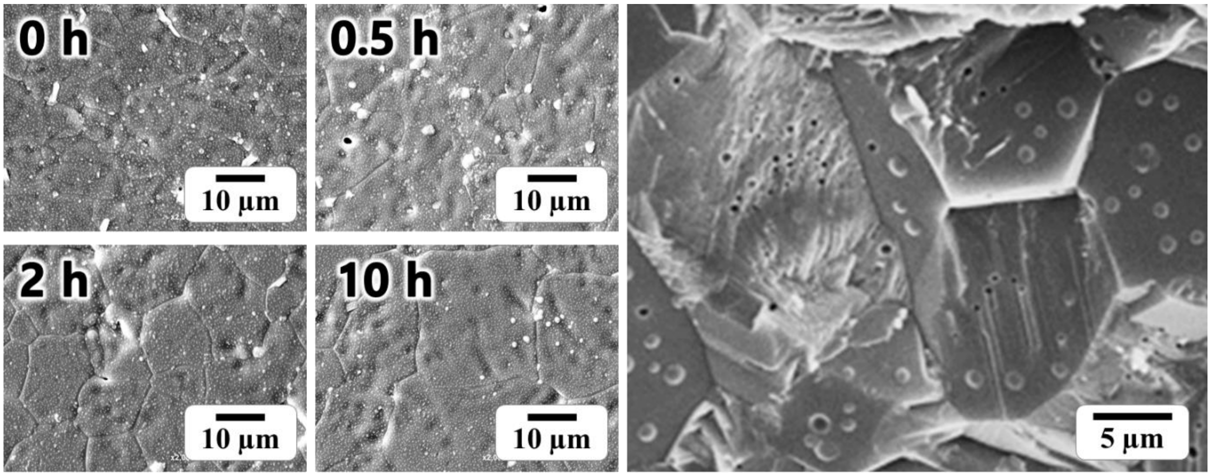

Figure S6. SEM images of TN-MgO sintered at 1400°C.

The specimens were polished and thermally etched to identify the grain sizes (left). The image on the right is an SEM micrograph showing the internal microstructure of the sintered body. In right SEM image, internal pores resulting from rapid grain growth are clearly observed [31].

Due to its relatively small quantity, this secondary phase is often not detectable by XRD. However, localized regions on the polished surface may exhibit white particles, which are secondary phases formed by the reaction between the donor and MgO. The amorphous phase, originally present as a liquid-like phase during sintering and composed of the donor and MgO, is known to reside along grain boundaries and internal pore surfaces [31]. Upon subsequent heat treatment at 1300 °C, this phase re-enters a liquid-like state, spreading over the polished surface. During cooling, it recrystallizes, resulting in the formation of droplet-like features visible on the surface. In addition, internal pores may undergo morphological distortion upon heat treatment at 1300 °C, as observed in the image, likely due to the presence and influence of the liquid-like phase on the surface.

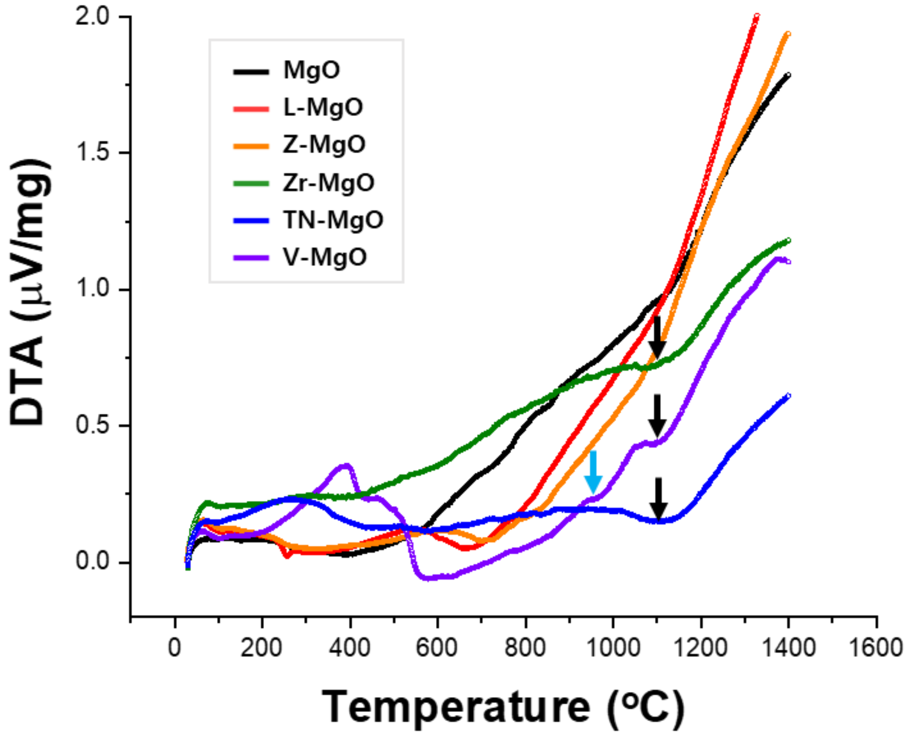

Figure S7. Differential thermal analysis (DTA) results in MgO, L-MgO, Z-MgO, MgO + Zr^{4+} (Zr-MgO), TN-MgO and V-MgO.

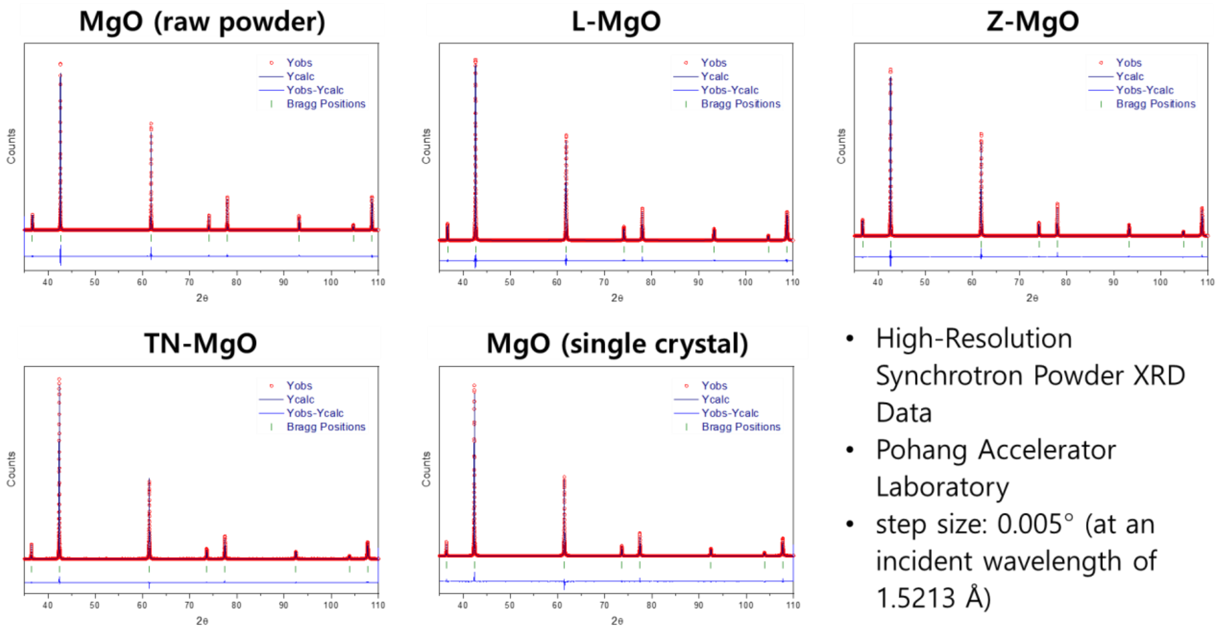

Specimen	Space group	*a* (Å)	Occupancy		Reliability factors				Occupancy difference[b)] (%)	Heat treatment temperature (℃)
			Mg	O[a)]	R_p (%)	R_{wp} (%)	R_{exp} (%)	*S*		
MgO (***raw powder***)		4.212046(7)	0.958(9)	1.00	9.31	13.1	8.72	1.50	-	-
MgO + **acceptor** *(Li^+)*		4.21185(2)	0.936(9)	1.00	6.71	9.37	5.96	1.57	**2.2**	1400
MgO + Zn^{2+}	*Fm-3m*	4.21209(2)	0.94(7)	1.00	7.86	10.4	6.03	1.72	**1.8**	1400
MgO + **donor** *(Ti^{4+}, Nb^{5+})*		4.21219(1)	0.896(3)	1.00	6.27	9.62	7.60	1.26	**6.2**	1400
MgO (***single crystal***)		4.21227(3)	0.868(7)	1.00	8.49	11.3	7.02	1.60	**9.0**	3000

[a)] *Fixed parameter*

[b)] *Occupancy difference:* Difference in Mg site occupancy relative to raw powder

Figure S8. Occupancy difference of Mg element in MgO materials prepared at various manufacturing temperatures. High-resolution powder diffractometer (HRPD) patterns and results of Rietveld refinement in various MgO materials.

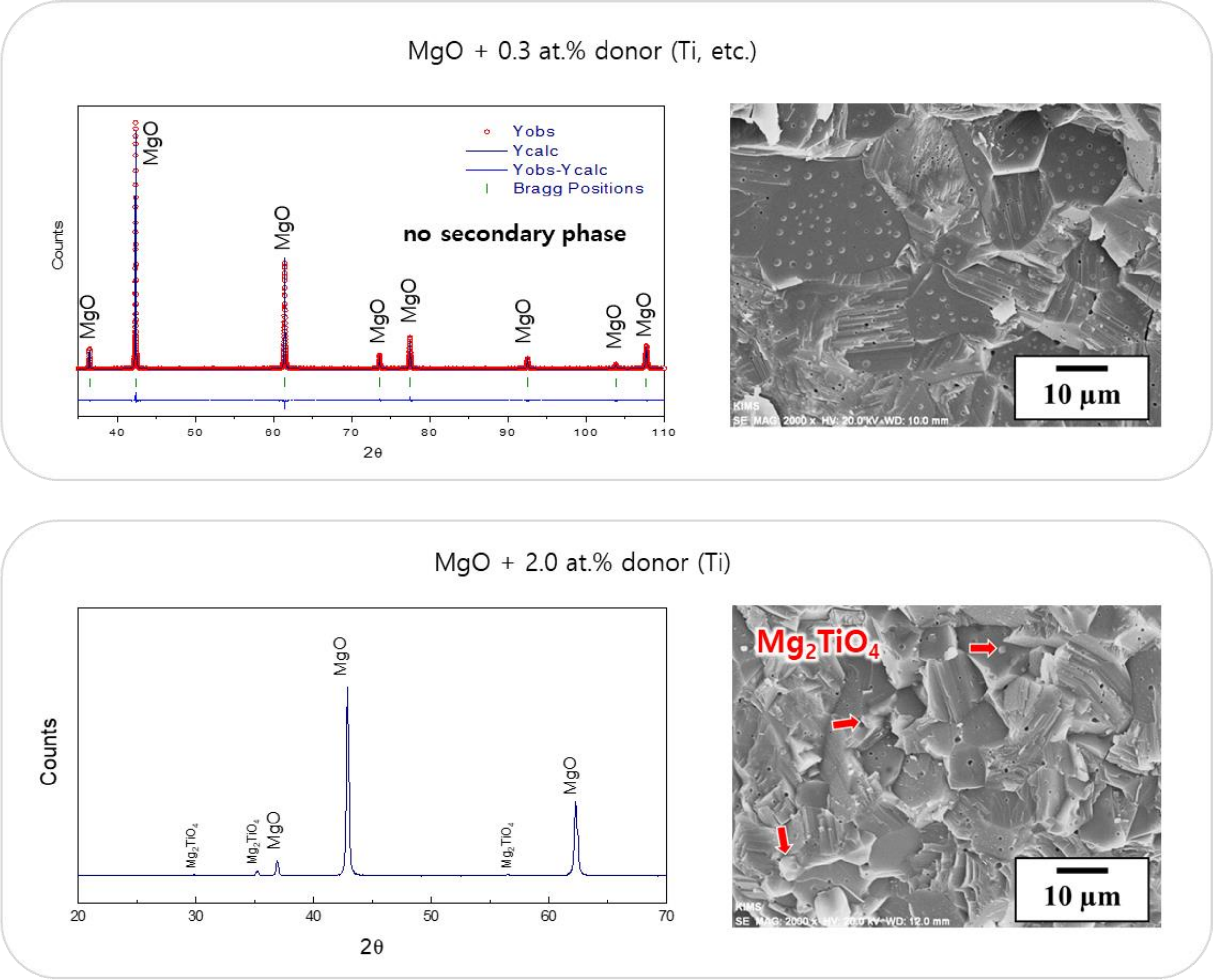

Figure S9. XRD patterns and SEM images of MgO + donors.

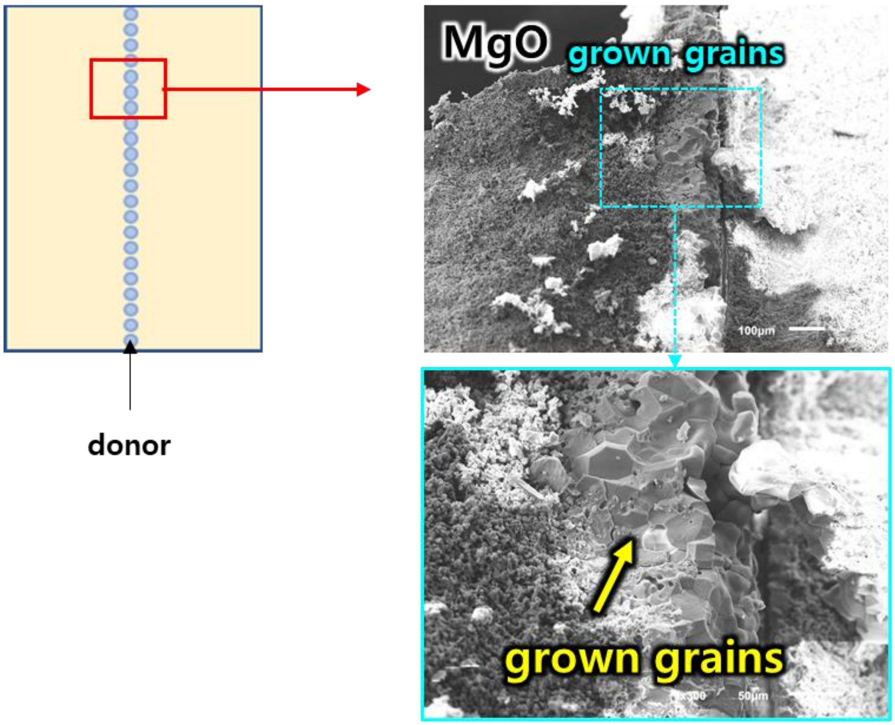

(a)

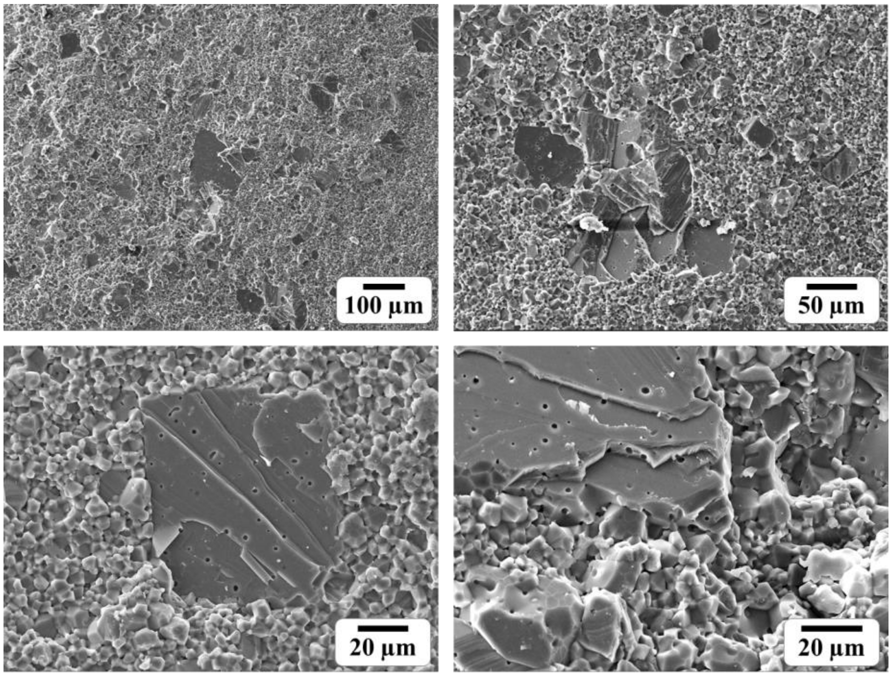

(b)

Figure S10. (a) Result of fundamental experiment to show local difference in MgO and (b) SEM images of d-MgO sintered at 1100 °C. AG is observed in the specimens.

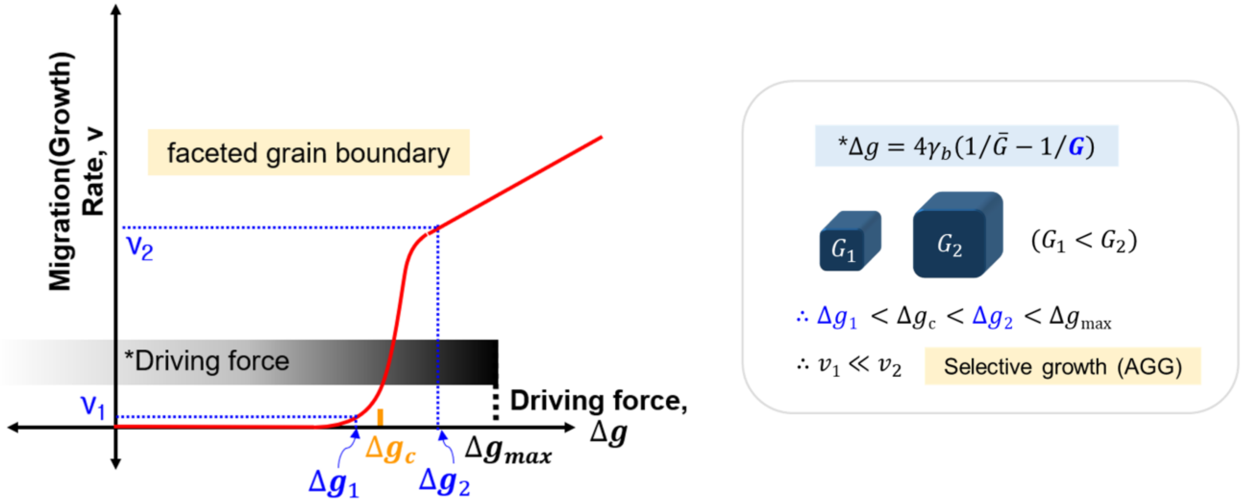

Figure S11. Schematic representation showing growth rate as a function of driving force for migration.

Abnormal grain growth (AGG) can occur with grain boundary pinning by solute segregation or secondary phase. Both mechanisms commonly result in a reduction of the driving force for grain growth. On the other hand, AGG has also been observed in pure systems where these drag effects are absent. From a fundamental standpoint, AGG behavior in pure systems has been explained by the grain boundary structure, which remains stable during sintering at high temperatures. Grain growth results from grain boundary migration. In the case of faceted grain boundaries, migration rate has been observed to be non-linear with respect to driving force for migration, as illustrated in Fig. 1(c). The non-linearity suggests the co-existence of two distinct kinetics of grain boundary migration, depending upon the driving force for migration (Δg*), as expressed by the equation 3, where *$\bar{G}$* is the mean grain size, *G* is the size of each grain and *γ_b* is the grain boundary energy. This equation describes the relationship between mean grain size in a polycrystalline system and the size of individual grains, providing a range of driving force for grain growth as shown by the bar in Fig. 1(c). The range extends from infinitely small value for the smallest grain to the maximum value of the largest grain (*Δg_{max}*) in the system. In the case of faceted grain boundaries, a significantly critical driving force (*Δg_c*) exists, dividing the system into two different grain growth mechanisms based on the *Δg* value. Relatively large grains with a higher driving force (*$\Delta g > \Delta g_c$*) exhibit significant growth rates (*v_1*) whereas smaller grains with lower driving forces (*$\Delta g < \Delta g_c$*) show very limited growth rates (*v_2*). This difference ultimately leads to selective grain growth, i.e. AGG as reported in many previous studies.*

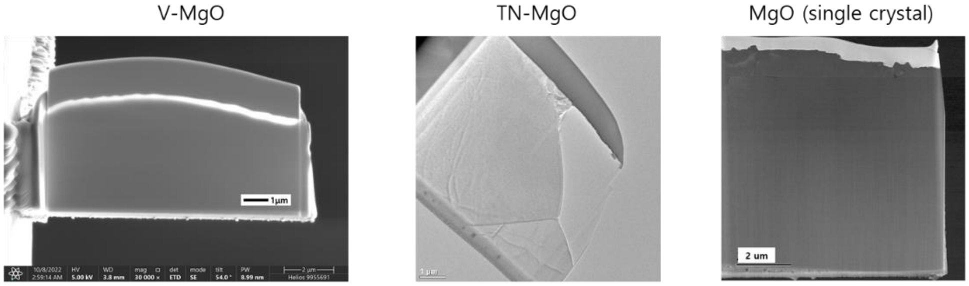

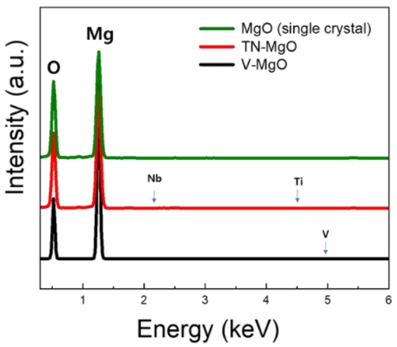

Elemental composition determined by TEM-EDS analysis

Element	O	Mg	V	Ti	Nb
MgO (single crystal)	52.55-55.29	44.71-47.45			
TN-MgO (1400℃ 2 h)	54.02-53.08	45.95-46.89		0.00-0.01	0.00-0.03
V-MgO (1200℃ 10 h)	50.66-51.84	48.14-49.33	0.00-0.02		
V-MgO (1100℃ 10 h)	50.10-50.80	49.20-49.88	0.00-0.01		

Figure S12. Donor concentrations detected within grains of LT-MgO samples by TEM analysis.

The concentration of Mg vacancies induced by donor doping can be inferred from the detected donor concentrations obtained through compositional analysis within MgO grain. For each sample, 8 to 26 measurement points were analyzed and compiled.

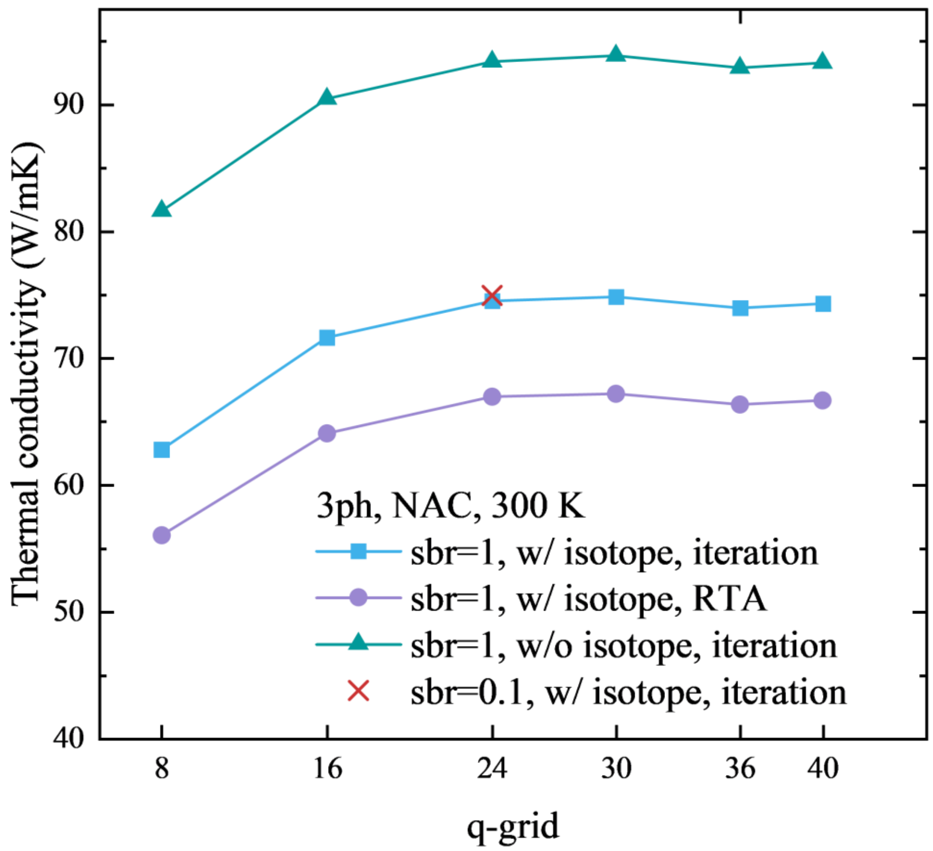

Fig. S13. q-mesh and Gaussian broadening factor (sbr) convergence test of thermal conductivity calculation.

"W/ isotope" indicates MgO containing naturally occurring isotopes of Mg and O. "W/o isotope" indicates MgO containing isotopically purified (or enriched) Mg and O. Thermal conductivity converges at 24×24×24 ***q****-mesh.*

Theoretical thermal conductivity of MgO:

To address the concern regarding the relatively high thermal conductivity (75–90 W/mK) we observed for MgO, we parameters, we find that the choice of exchange-correlation functional has the most significant impact on the predicted value while other factors including phonon renormalization and four-phonon scattering plays marginal role at room temperature. Dekura and Tsuchiya have also found sensitivity of thermal conductivity with respect to lattice constant, which is in turn determined by exchange-correlation functionals. Given the sensitivity of first-principles thermal conductivity calculations to methodological choices, it is not appropriate to treat any single value in the literature—including our own—as the definitive theoretical thermal conductivity of MgO. It is also important to note that theoretical predictions may not represent

the upper limit of thermal conductivity. For example, even for benchmark materials as well-studied as isotopically pure silicon, first-principles calculations—regardless of the exchange-correlation functional used—typically yield values between 137–145 W/mK, while the experimental value is about 153 W/mK. This corresponds to a systematic underprediction of approximately 5–10%. In light of this, and supported by both our calculations and the range of values reported in the literature, we conclude that thermal conductivities in the range of 75–90 W/mK for MgO are plausible and cannot be ruled out.

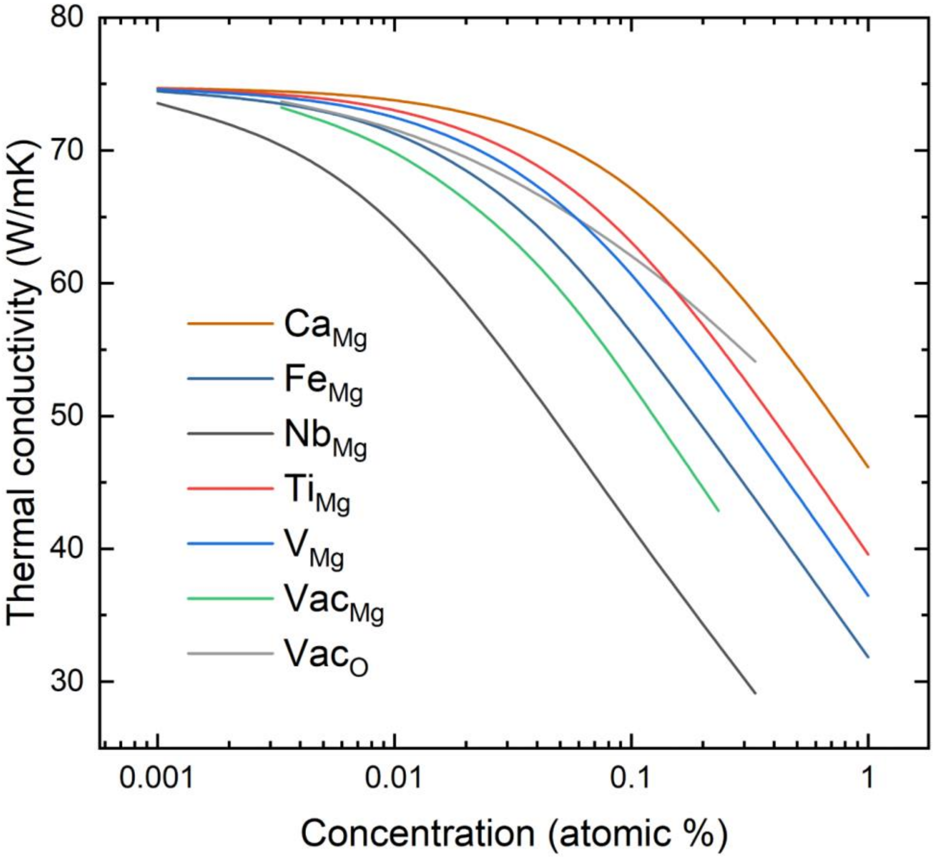

Fig. S14. Thermal conductivity of MgO as a function of defect concentration at room temperature calculated using Boltzmann transport equation from first principles.

Thermal conductivity of MgO with defects

We have also quantitatively estimated the reduction in thermal conductivity of MgO caused by common point defects, including magnesium vacancy (Vac_{Mg}), oxygen vacancy (Vac_{O}), calcium substitution of magnesium (Ca_{Mg}), and iron substitution (Fe_{Mg}). The phonon defect scattering rates are calculated using Tamura's formalism extended by Klemens, which accounts for both mass and bond changes caused by defects.[11-13] Figure S14 shows the reduction of thermal conductivity as a function of defects concentration. With a moderate defect concentration of 0.01% to 0.1%, the thermal conductivity can be reduced to 50-60 W/mK. Grain boundaries could be another source of low thermal conductivity in literature since the phonon mean free path in MgO can extend to 10 µm (Fig. S15) – grain size smaller than ~100 µm would reduce the thermal conductivity of MgO.

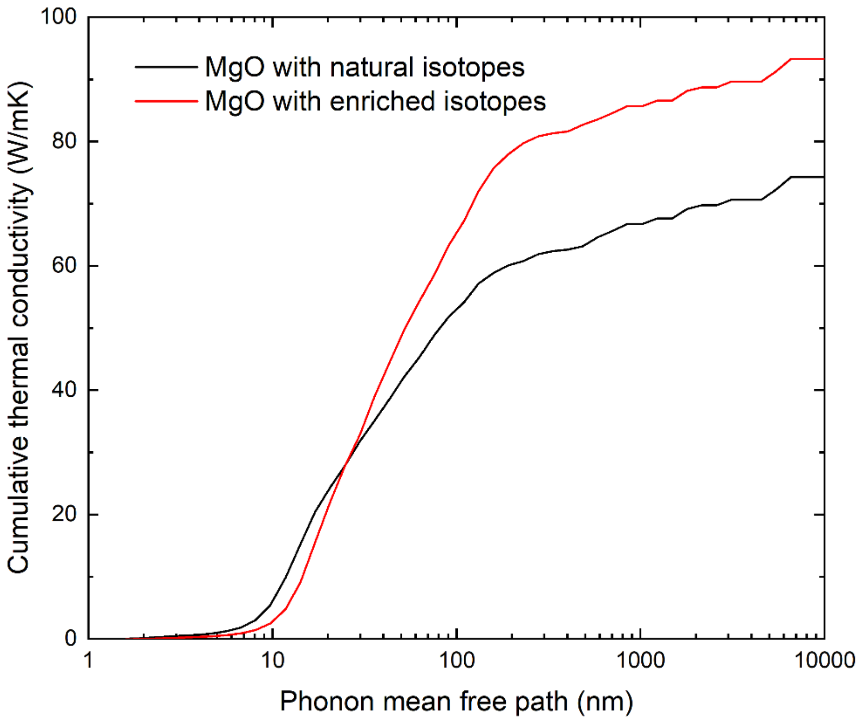

Fig. S15. Cumulative thermal conductivity as a function of phonon mean free path calculated by first principles.

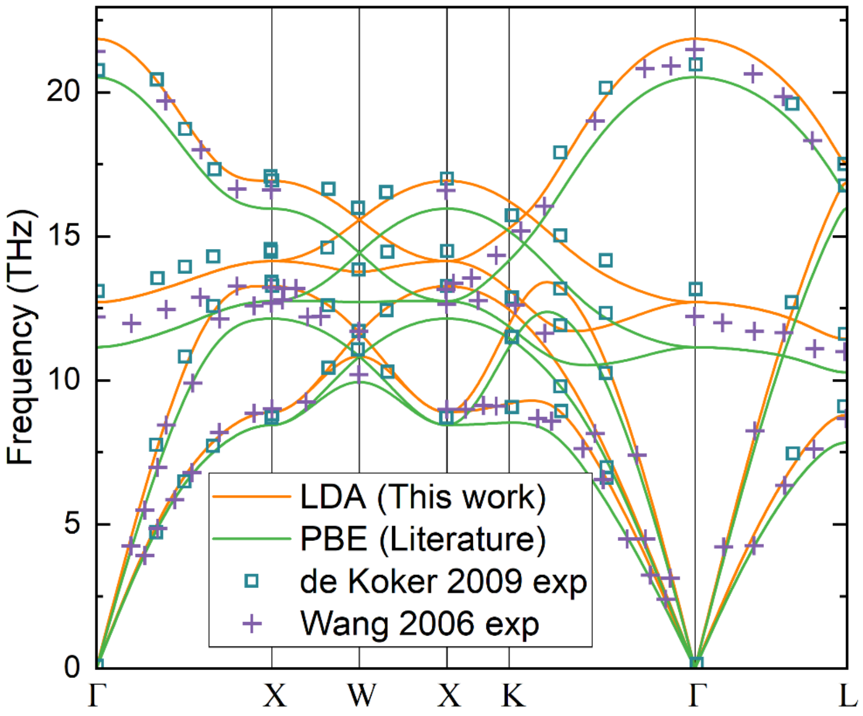

Fig. S16. Phonon dispersion of MgO calculated by first principles compared to experimental data as well as the literature calculations using PBE exchange correlation functional. Refs: PBE. de Koker 2009. Wang 2006.[14-16]

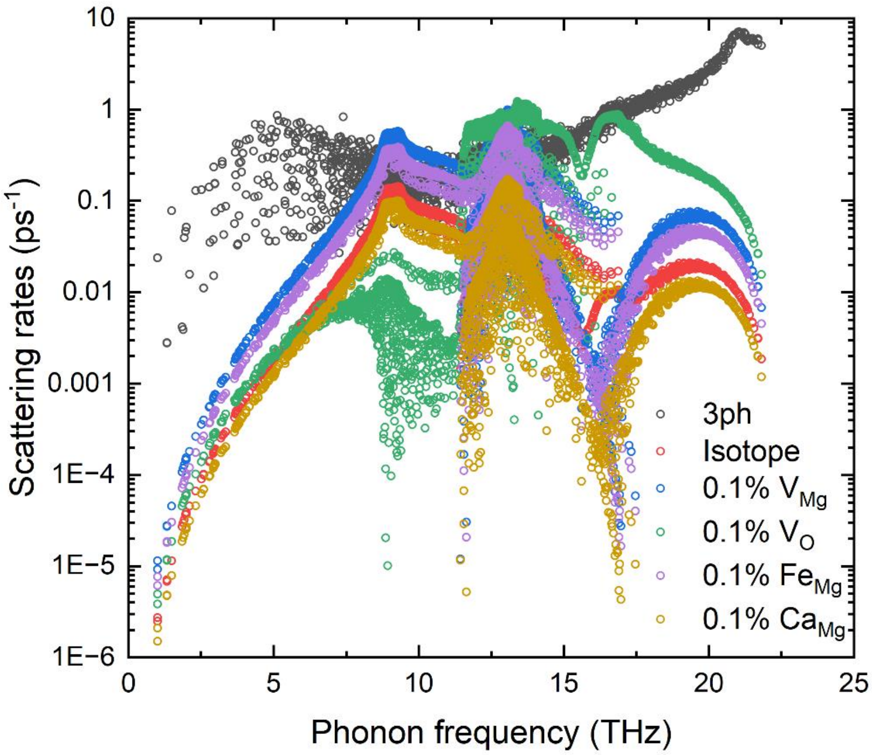

Fig. S17: Three-phonon, phonon-isotope, and phonon-point defects scattering rates calculated by first principles.

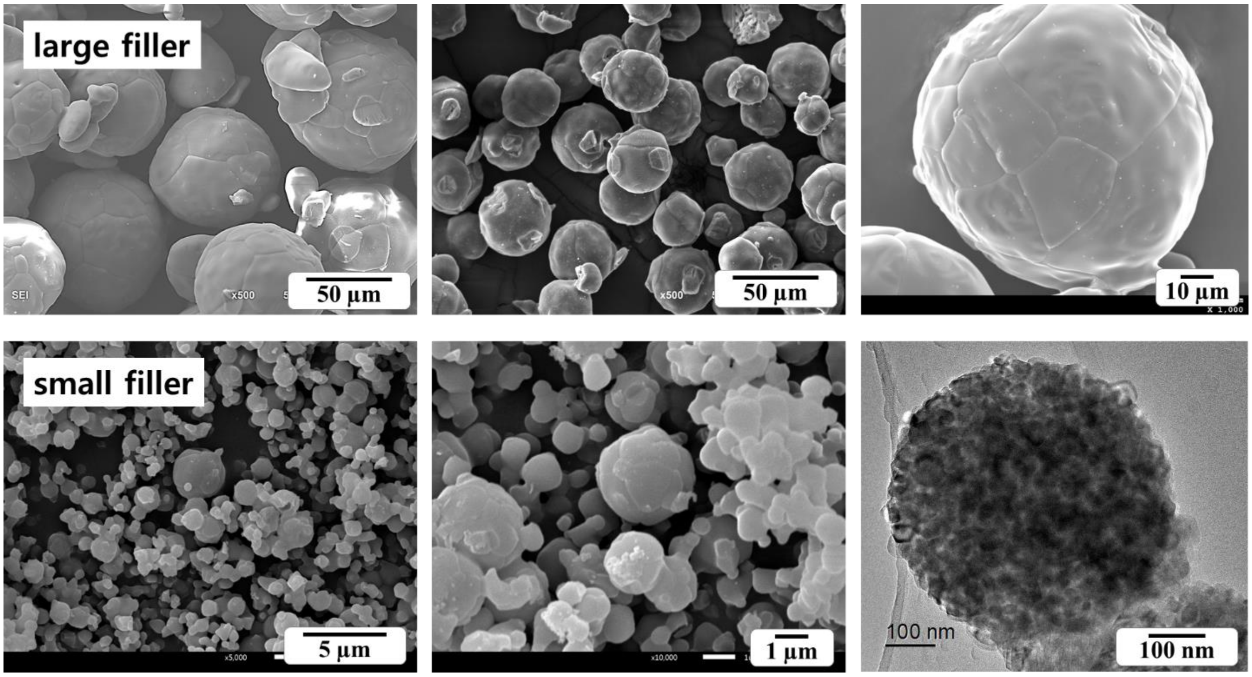

Figure S18. SEM and TEM images of various LT MgO fillers.

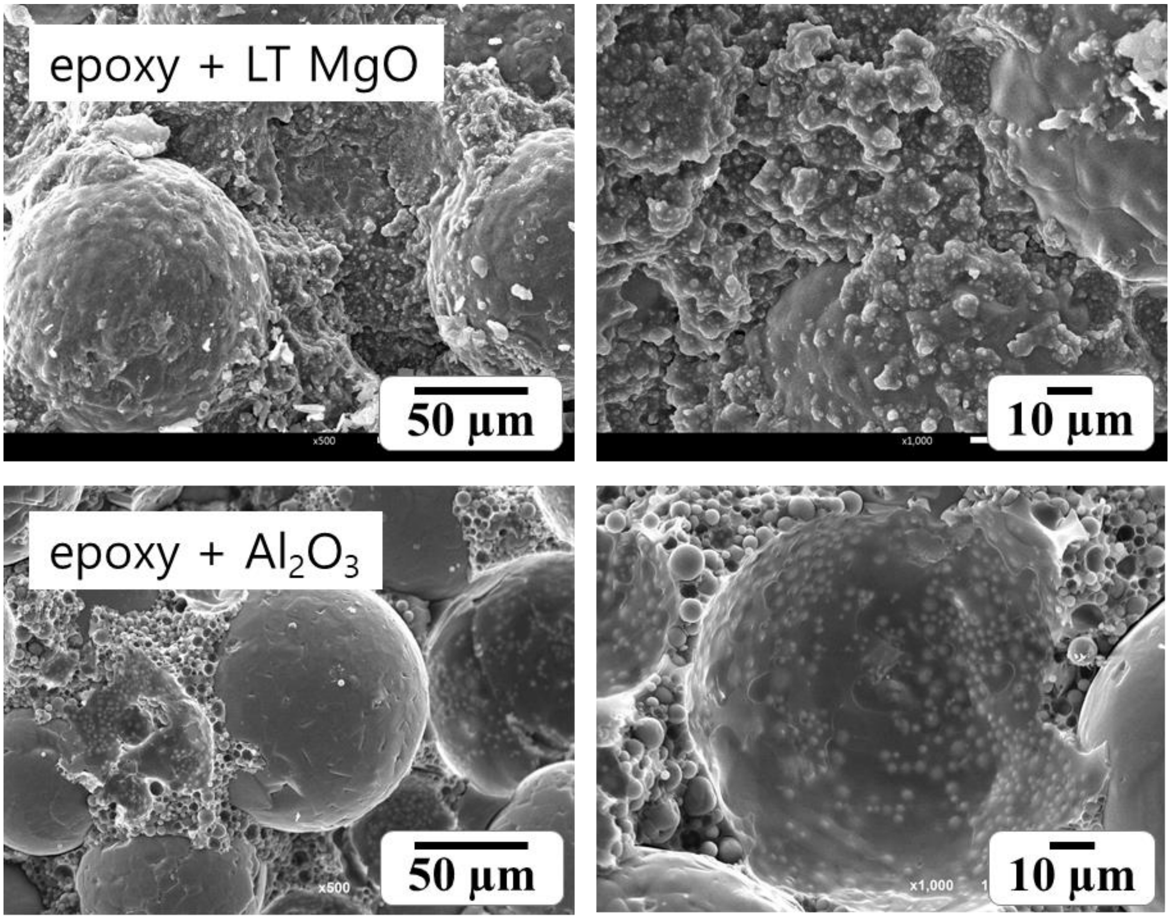

Figure S19. SEM images of epoxy + LT MgO filler and epoxy + Al_2O_3 filler.

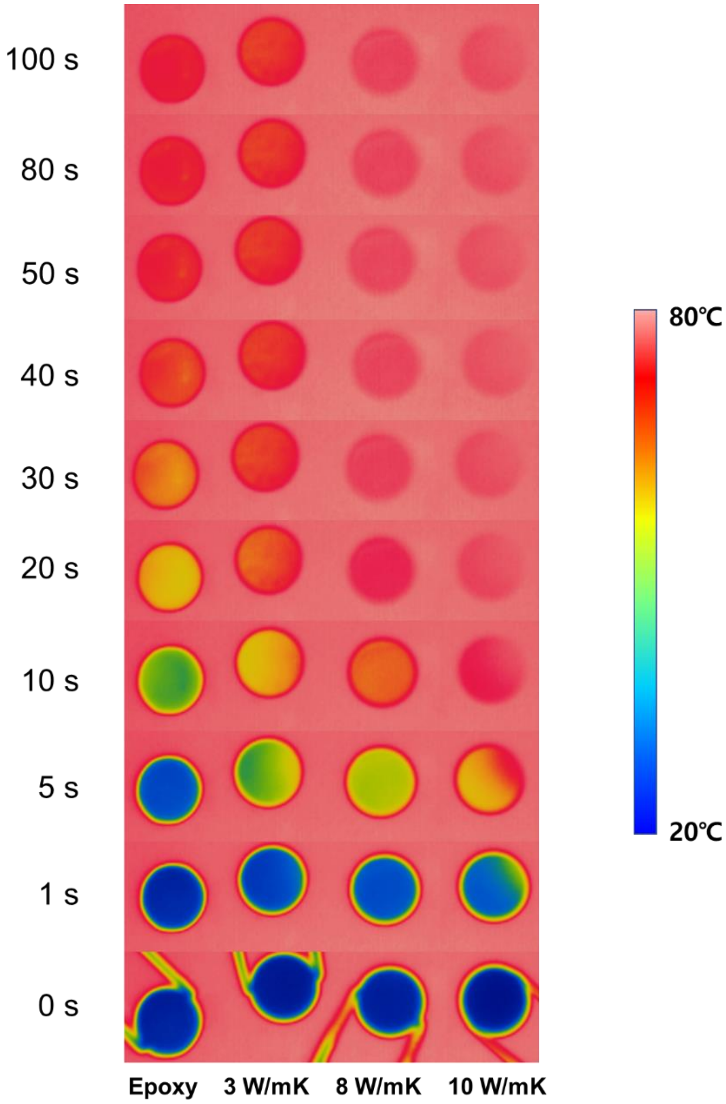

Figure S20. IR images of heat transfer rates in various TIMs.

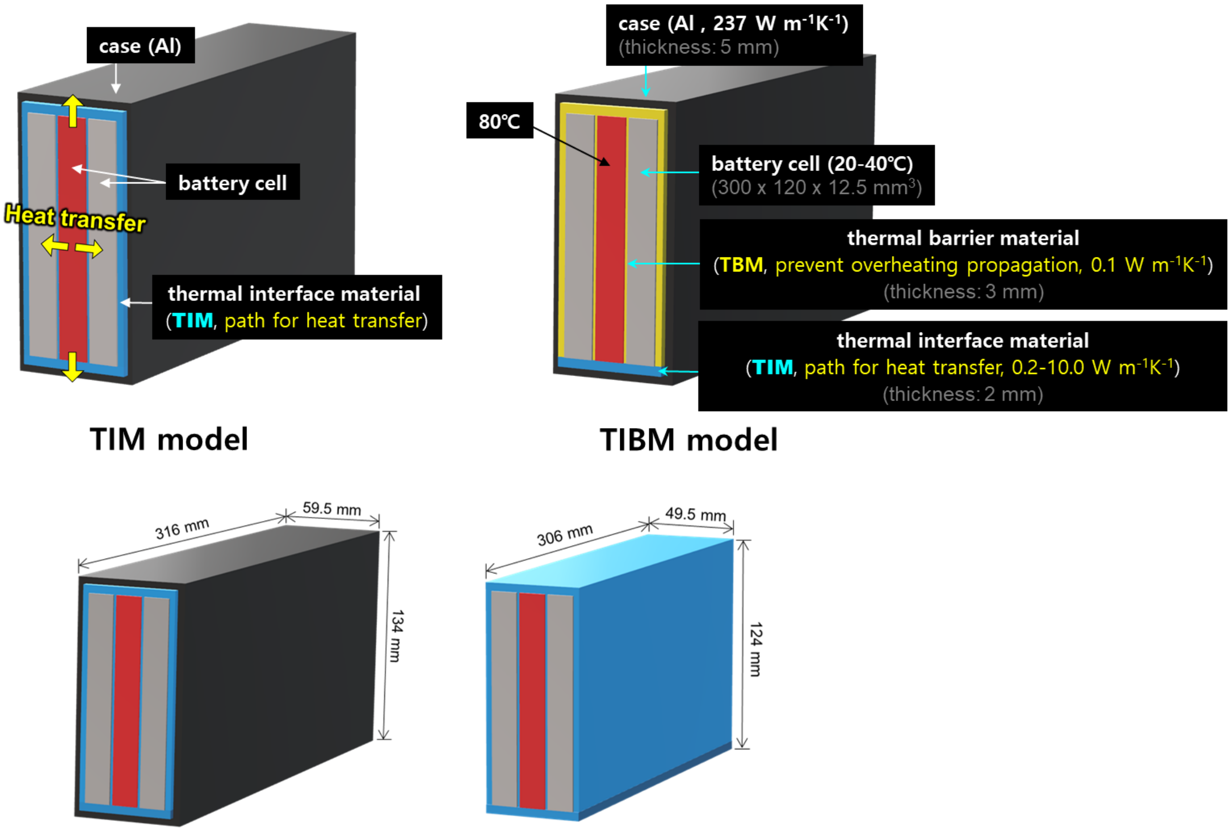

Figure S21. Structure of battery modules (TIM and TIBM models) for simulation. Two types of battery packages are currently used in EVs.

TBM is used to protect the high temperature of a suddenly hot cell from being transferred to neighbor cells. In general, batteries in electric vehicles are operated in the 30-40 ℃ temperature range, so this study assumes a room temperature of 20 ℃ and operating temperatures of 30 ℃ and 40 ℃ for comparison. The thermal conductivity of TIMs was compared between the polymer without thermal ceramic filler and with commercial filler or LT MgO filler. The cell size specifications are based on high-quality package-products currently used in EVs.

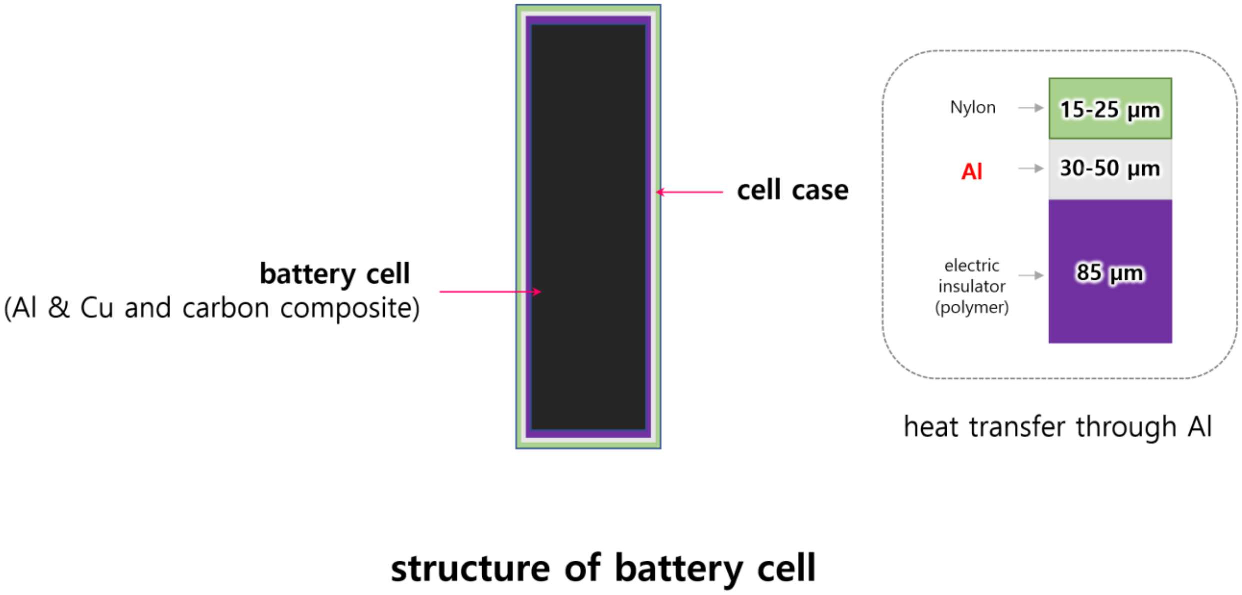

Figure S22. Internal details of battery cell.

The battery cell is mainly composed of metals (Al and Cu) and carbon composite, and the heat can be transferred through the Al foil of a cell case. However, the cell case contains some polymer layers. Therefore, the various thermal conductivities (0.25-200 W m^{-1} K^{-1}, in this study) need to be considered and calculated in the cell simulation.

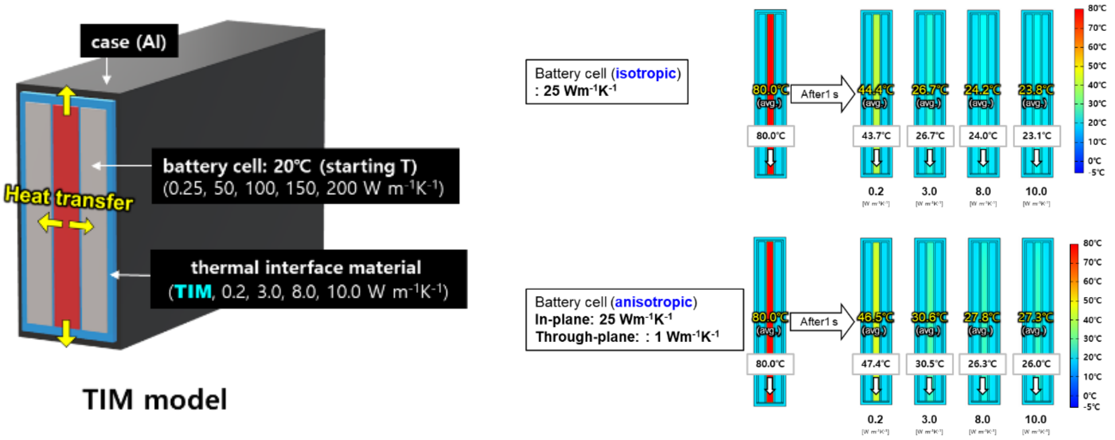

(a)

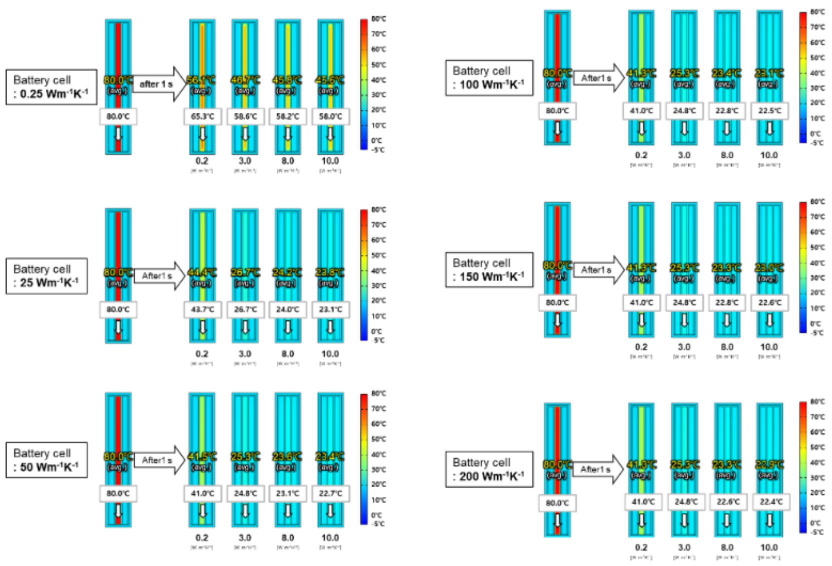

(b)

Figure S23. Cooling performance of a battery cell as a function of its intrinsic thermal conductivity.

(a) Comparative analysis of isotropic versus anisotropic thermal conduction.

(b) Cooling performance data organized as a function of thermal conductivity.

The thermal conductivity of battery cells is known to be anisotropic, with reported values of approximately 20–30 W·m⁻¹·K⁻¹ in the in-plane direction and 0.2–2 W·m⁻¹·K⁻¹ in the through-plane direction.[7–8] Accordingly, as illustrated in Figure S23(a), we assumed an in-plane thermal conductivity of 25 W·m⁻¹·K⁻¹ and a through-plane thermal conductivity of 1.0 W·m⁻¹·K⁻¹, and compared this anisotropic case with the isotropic case of 25 W·m⁻¹·K⁻¹. The cooling behavior of the two models did not differ significantly Except for the reported case of 25 W·m⁻¹·K⁻¹ versus 1

$W{\cdot}m^{-1}{\cdot}K^{-1}$, there is little documentation of directional thermal conductivities, making it difficult to unambiguously specify anisotropic differences when assuming other thermal conductivity values. Therefore, for all other thermal conductivity values considered in this study, an isotropic assumption was adopted in the calculations.

To examine the effects of thermal insulation and high-performance heat-spreading materials, extreme cases were also considered, including a low thermal conductivity of 0.25 $W\,m^{-1}\,K^{-1}$ (representing poor thermal dissipation) and the thermal conductivity of aluminum (237 $W\,m^{-1}\,K^{-1}$). Thus, under the hypothetical scenario where conventional thermal insulation materials used in cells are replaced with advanced heat-dissipating materials, a thermal conductivity as high as 200 $W\,m^{-1}\,K^{-1}$ was assumed. Notably, recent studies have reported that the effective in-plane thermal conductivity of battery cells has been enhanced up to 141 $W\,m^{-1}\,K^{-1}$.[9]

As seen in Figure S23(b), the variation in cooling behavior is negligible across the range of thermal conductivities examined, except at 0.25 $W\,m^{-1}\,K^{-1}$, which results in a marked deviation. However, considering the representative thermal conductivities of constituent materials in typical battery cells (see Fig. S19), values as low as 0.25 $W\,m^{-1}\,K^{-1}$ are not realistic.

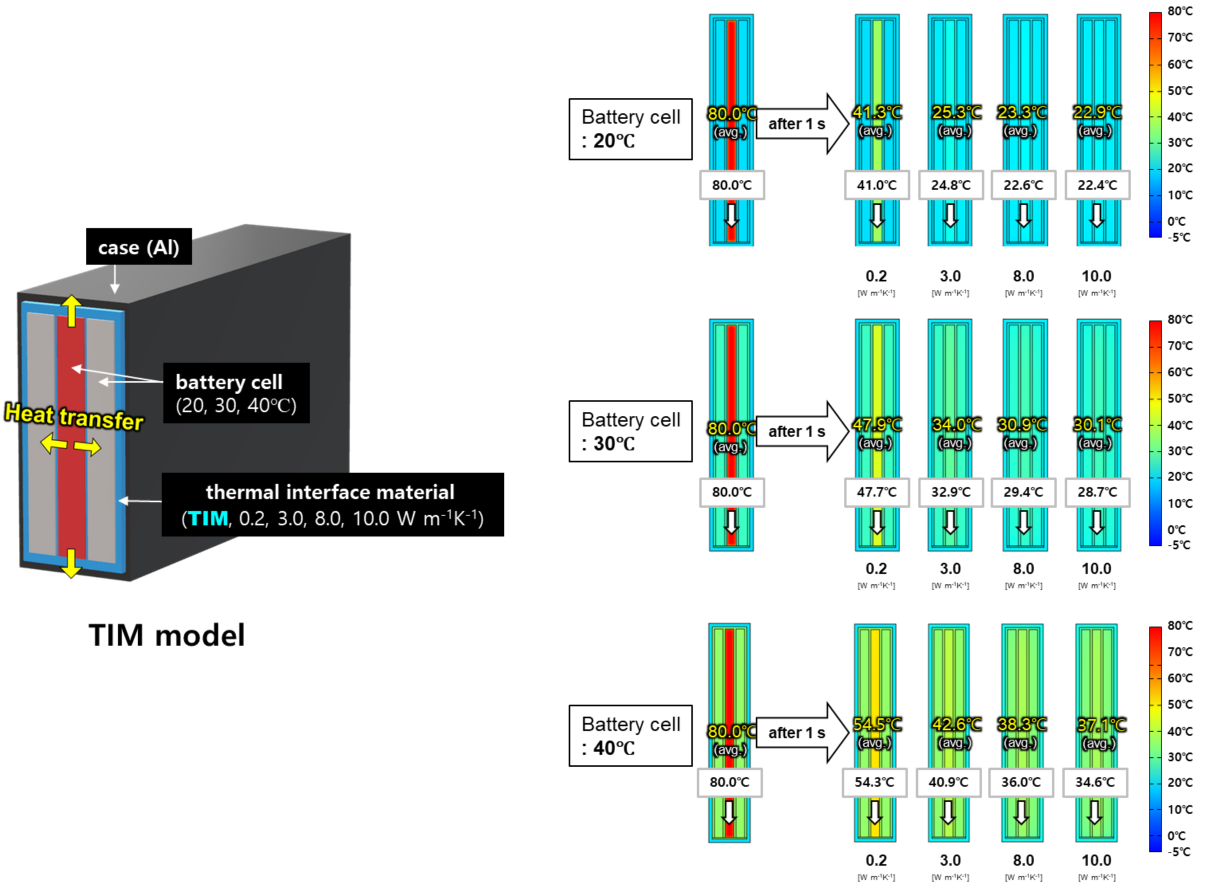

Figure S24. Differences of cooling performance depending on temperature of battery cell.

In general, batteries in electric vehicles are operated in the 30-40℃ temperature range. Thus, the cell temperatures were assumed to be 20℃ (room temperature) and 30℃ & 40℃ (the operating temperatures of a battery cell), for comparison. The higher the temperature of the cell was, the better the improvement of cooling performance due to the thermal conductivity of the TIM was. The higher the operating temperature of the cell is, the more the temperature of the cell can suddenly rise to 80°C. Thus, the high thermal conductivity of the TIM is critical for EV fire prevention.

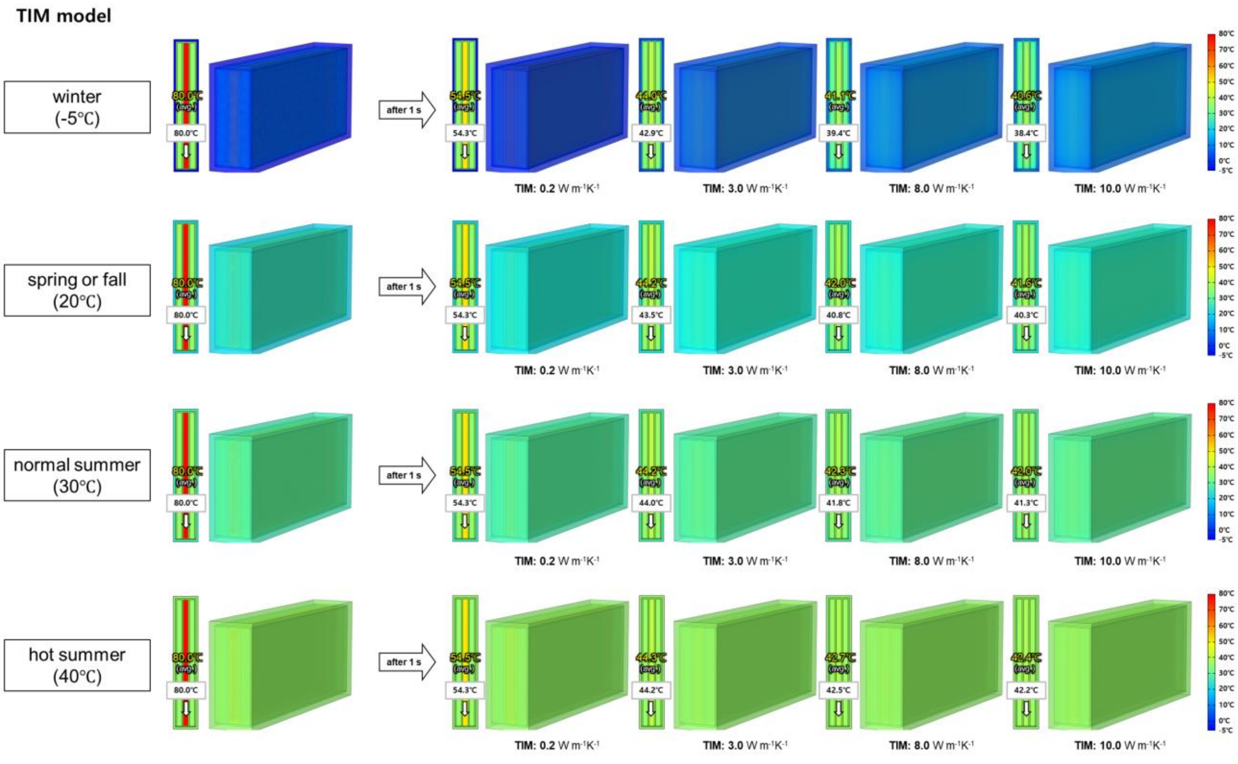

Figure S25. Differences of cooling performance depending on air temperature in TIM model.

Compared to hot summer, the cooling performance improved by the high thermal conductivity of TIM was observed in cold winter.

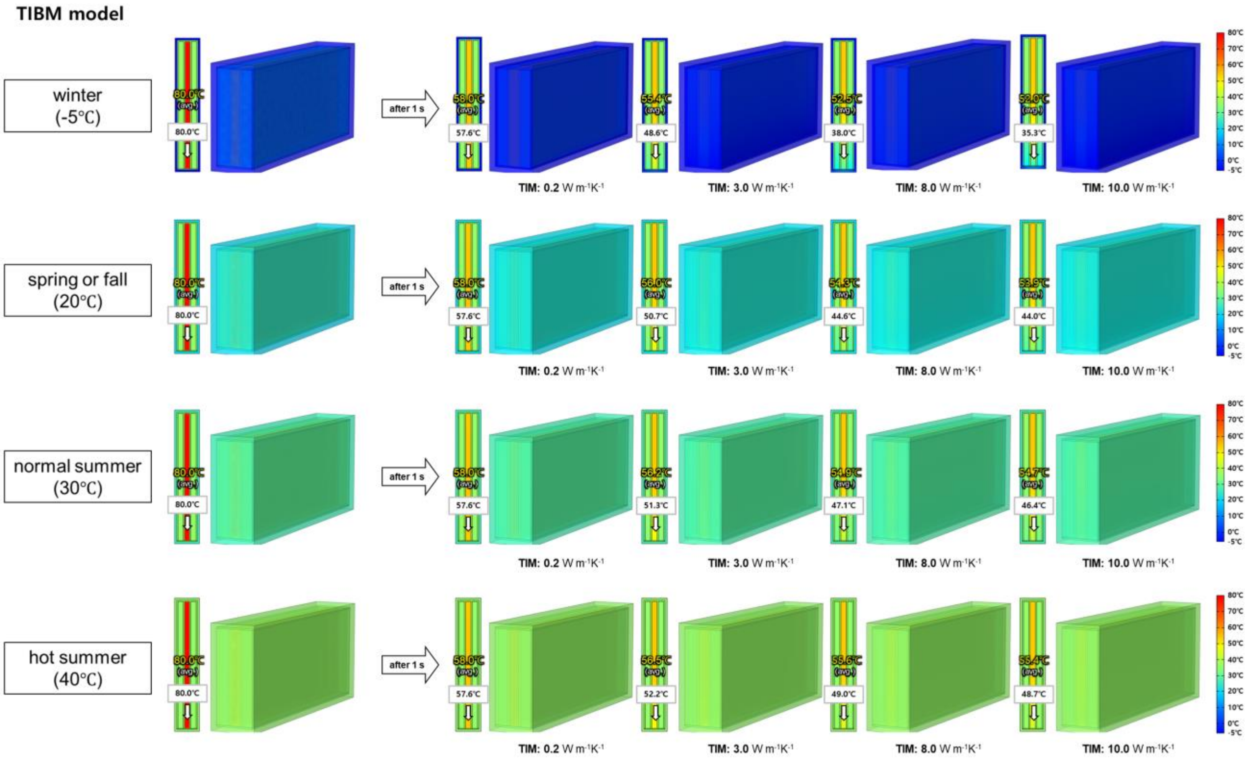

Figure S26. Differences of cooling performance depending on air temperature in TIBM model.

The temperature of the bottom area (near TIM) cooled down quickly, while the average temperature of the cell cooled down relatively slowly. Compared to TIM model, While the TIBM model is excellent at preventing the high temperatures of dangerous cells from being transferred to neighbors, it can sacrifice on the performance of cooling hot cells. Compared to hot summer, the cooling performance improved by the high thermal conductivity of TIM was observed in cold winter.

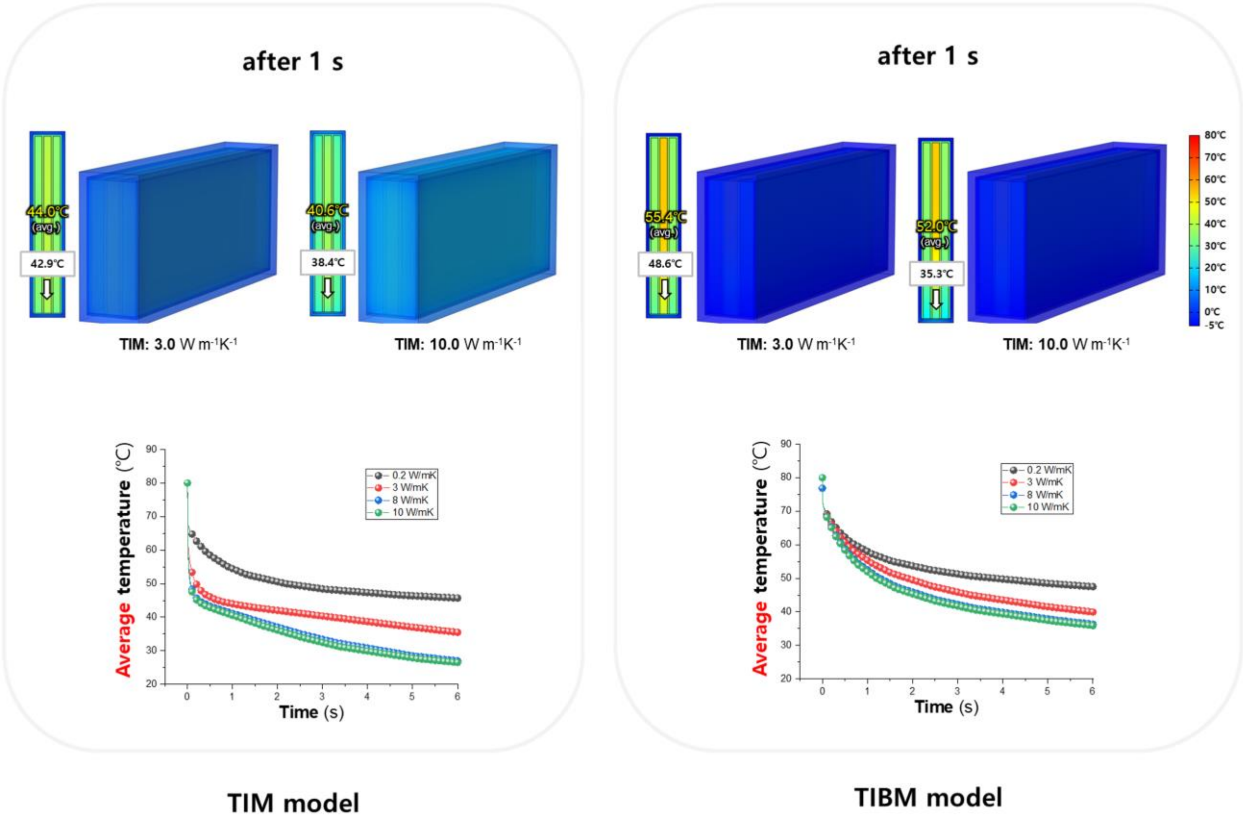

Figure S27. Differences of cooling performance depending on thermal conductivity of TIM in TIM and TIBM models.

In TIBM model, the bottom temperature varied significantly with the thermal conductivity of the TIM, but the significant differences were not found in the average temperature. Most of all, the average temperature drops quickly in TIM model, while it drops relatively slowly in TIBM model. On the contrary, the two models do not show the significant difference at the bottom temperature.

Table S1. Overview of thermal conductivity performance for various MgO materials, categorized by sintering method, temperature, cost, and inherent limitations.

**sintering method**	**sintering temperature** (℃)	**TC** (W m^{-1} K^{-1})	**cost**	**limitation**	**ref**
simple sintering	*1100*	***70***	*low*	-	*this study*
simple sintering	*1200*	***>80***	*low*	-	*this study*
simple sintering	860	24	low	low TC	[39]
simple sintering	1400	54	low	low TC	[30]
simple sintering	1400	55	low	low TC	[31]
simple sintering	1600	47	medium	high sintering temperature	[40]
simple sintering	1700	<34	medium	high sintering temperature & low TC	[41]
vacuum sintering	1300	47	high	vacuum sintering	[42]
vacuum sintering	1800	52	high	vacuum sintering	[43]
vacuum sintering	1800	32	high	vacuum sintering & low TC	[43]
hot isostatic pressing	1300	18	very high	high processing cost & low TC	[44]
hot isostatic pressing	1600	54	very high	high processing cost & low TC	[45]
vacuum sintering + hot isostatic pressing	1650	53	very high	high processing cost & low TC	[46]
hot isostatic pressing under Ar	1650	55	very high	high processing cost & low TC	[47]
hot-press sintering	1250	<17	very high	high processing cost & low TC	[48]

The reference numbers used in this table correspond to those cited in the main manuscript, not the Supporting Information.

Table S2. Comparative table of thermal reactions in lithium-ion batteries.[10]

**Temperature (°C)**	**Reaction Stage**	**Behavior Summary**
**60–80**	Onset of SEI decomposition	Decomposition of the solid electrolyte interphase begins, generating gases and minor heat.
**80–100**	Electrolyte breakdown	Combustible electrolyte components start to degrade; heat generation increases, requiring close monitoring.
**~135**	Separator melting (PE/PP)	Melting of polyethylene/polypropylene separators begins, risking structural collapse and internal short circuits (ISCs).
**110–140**	Gas venting	Excessive internal pressure triggers activation of safety vent; thermal management may delay this event.
**140–150**	Thermal runaway onset (T_{TR})	Critical temperature range where electrolyte combustion and ISCs may initiate uncontrollable exothermic reactions.
**>150**	Rapid exothermic chain reactions	Cathode decomposition, electrolyte oxidation, and oxygen release lead to rapid temperature escalation and full thermal runaway; passive cooling becomes ineffective.

The thermal behavior of lithium-ion batteries is governed by a sequence of temperature-dependent reactions that can lead to hazardous conditions if not properly managed. At moderate temperatures of approximately ***60–80°C****, the* ***solid electrolyte interphase (SEI)*** *begins to decompose, releasing heat and gaseous byproducts. Under ideal thermal management, this early-stage reaction can be stabilized without escalation.*

Between ***80–100°C****, the* ***electrolyte undergoes decomposition****, further contributing to internal pressure buildup and heat generation. Although this stage may still be controllable with adequate cooling, the system becomes increasingly unstable.*

As temperatures exceed 100°C, separator melting (~130–140°C) and gas venting (115–140°C) occur, increasing the risk of internal short circuits and pressure buildup. Thermal runaway typically initiates between 130–150°C, beyond which exothermic reactions rapidly escalate, and cooling measures become largely ineffective.

Therefore, the cooling performance of battery packs is more critical in the ***≤80°C*** *regime than at higher temperatures.*

Table S3. Sintering behavior in MgO materials with various additives.

Element of additive	Kind	Lowest eutectic point (LEP, °C)	Sintering at ≤1,400°C	Sintering behavior
**Li**	acceptor	1,423	not sintered	normal sintering behavior (LEP: higher than 1,400°C)
**Zn**	-	1,808	not sintered	normal sintering behavior (LEP: higher than 1,400°C)
**Ti**	*donor*	*1,605*	*well-sintered*	*donor effect* *(LEP: higher than 1,400°C)*
**Nb**	*donor*	*1,430*	*well-sintered*	*donor effect* *(LEP: higher than 1,400°C)*
**B**	donor	1,146	well-sintered	normal sintering behavior (LEP: lower than 1,400°C)
**V**	donor	604	well-sintered	normal sintering behavior (LEP: lower than 1,400°C)

Table S4: Theoretically predicted thermal conductivity of MgO based on first-principles density functional theory (DFT) calculations.

Year	Methods	Thermal conductivity of MgO at room temperature	References
**2009**	DFT, LDA, BTE, RTA (first principles molecular dynamics)	48.5 W $m^{-1}K^{-1}$	17
**2010**	DFT, LDA, ab initio nonequilibrium molecular dynamics	55 W $m^{-1}K^{-1}$	18
**2010**	DFT, LDA, BTE, RTA (3ph)	66 W $m^{-1}K^{-1}$ w/ natural isotopes<br>96 W $m^{-1}K^{-1}$ w/ enriched isotopes	19
**2017**	DFT, LDA, full BTE (3ph)	54-67 W $m^{-1}K^{-1}$	20
**2018**	DFT, PBE, ab initio Green-Kubo molecular dynamics	60 W $m^{-1}K^{-1}$	21
**2020**	DFT, PBEsol, full BTE (3ph)	48 W $m^{-1}K^{-1}$	22
**2020**	DFT, PBEsol, full BTE (3+4ph), phonon renormalization	53 W $m^{-1}K^{-1}$	22
**2023**	DFT, PBE, full BTE (3ph)	50 W $m^{-1}K^{-1}$	23
**2023**	DFT, PBE, full BTE (3+4ph), temperature dependent force constants	46 W $m^{-1}K^{-1}$	23
**2025**	DFT, LDA, full BTE (3ph), converged **q**-mesh	75 W $m^{-1}K^{-1}$ w/ natural isotopes<br>94 W $m^{-1}K^{-1}$ w/ enriched isotopes	This study

Most studies compute the thermal conductivity by solving the Boltzmann transport equation (BTE) using either the relaxation time approximation (RTA) or a full iterative approach. The exchange-correlation functionals used in each work are specified, including the local density approximation (LDA), Perdew–Burke–Ernzerhof (PBE), and PBE for solids (PBEsol). Parentheses in the "Method" column indicate how the phonon relaxation time was obtained in the calculation.

Table S5. Structural dimensions and thermophysical properties of materials utilized in thermal simulation model.

	**thickness** (mm)	**size** (mm x mm x mm)	**thermal conductivity** (W m^{-1} K^{-1})	**density** (kg m^{-3})	**specific heat** (J kg^{-1} K^{-1})
**aluminum case**	5	316 x 134 x 59.5	237	2000	1000
**battery cell**		300 x 120 x 125	25 *(in-plane)*<br>1 *(through-plane)*	2700	904
**TBM**	3		0.1	1050	1200
**TIM**	2	306 x 124 x 49.5	0.2	1200	1100
			3.0	2340	904
			8.0	2920	1072
			10.0	3004	982

References

1. Konar, B., Kim, D.-G. & Jung, I.-H. Coupled phase diagram experiments and thermodynamic optimization of the binary Li_2O-MgO and Li_2O-CaO systems and ternary Li_2O-MgO-CaO system, *Ceram. Int.* **2017**, *43*, 135055-13062. https://doi.org/10.1016/j.ceramint.2017.06.143
2. Segnit, E. R. & Holland, A. E., The system MgO-ZnO-SiO_2, *J. Am. Ceram. Soc.* **1965**, *48* 409-413. https://doi.org/10.1111/j.1151-2916.1965.tb14778.x
3. Mutluer, T. & Timucin, M., Phase equilibria in the system MgO-B_2O_3, *J. Am. Ceram. Soc.* **1975,** *58*, 196-197. https://doi.org/10.111/j.1151-2916.1975.tb11442.x
4. Eriksson, G. & Pelton, A. D., Critical evaluation and optimization of the thermodynamic properties and phase diagrams of the MnO-TiO_2, MgO-TiO_2, FeO-TiO_2, Ti_2O_3-TiO_2, Na_2O-TiO_2, and K_2O-TiO_2 systems, *Metall. Trans. B*, **1993**, 24, 795-805. https://doi.org/10.1007/BF02663140
5. Abbattista, F. A., Rolando, P. & Grassi G. B., On system MgO-Nb_2O_5, *Ann. Chem.* **1970**, *60* 426-435.
6. Kerby, R. C. & Wilson, J. R., Wilson, Solid-liquid phase equilibria for the ternary systems V_2O_5-Na_2O-Fe_2O_3, V_2O_5-Na_2O-Cr_2O_3, and V_2O_5-Na_2O-MgO, *Can. J. Chem.* **1973**, *51* 1032-1040. https://doi.org/10.1139/v73-153
7. Drake, S. J., Wetz, D. A., Ostanek, J. K., Miller, S. P., Heinzel, J. M. & Jain, A., Measurement of anisotropic thermophysical properties of cylindrical Li-ion cells, J. Power Sources **2014**, 252, 298–304. https://doi.org/10.1016/j.jpowsour.2013.11.051
8. Bandhauer, T. M., Garimella, S. & Fuller, T. F., A critical review of thermal issues in lithium-ion batteries, J. Electrochem. Soc. 2011, 158, R1. https://doi.org/10.1149/1.3515880
9. Koo, B., Goli, P., Sumant, A. V., dos Santos Claro, P. C., Rajh, T., Johnson, C. S., Balandin, A. A. & Shevchenko, E. V., Toward lithium ion batteries with enhanced thermal conductivity, *ACS Nano* **2014**, *8*(7), 7202–7207. https://doi.org/10.1021/nn501081p
10. Feng, X., Ouyang, M., Liu, X., Lu, L., Xia, Y. & He, X., Thermal runaway mechanism of lithium ion battery for electric vehicles: A review, *Energy Storage Mater.* **2018**, *10*, 246–267. https://doi.org/10.1016/j.ensm.2017.05.013
11. Callaway, J., von Baeyer, H.C., Effect of point imperfections on lattice thermal conductivity, Phys. Rev. B 1983, 27, 858. https://doi.org/10.1103/PhysRevB.27.858
12. Klemens, P.G., The scattering of low-frequency lattice waves by static imperfections, Proc. Phys. Soc. A 1955, 68, 1113–1128. https://doi.org/10.1088/0370-1298/68/12/303
13. Feng, T., Ruan, X., Prediction of spectral phonon mean free path and thermal conductivity with applications to thermoelectrics and thermal management: a review, J. Nanomater. 2014, 2014, 206370. https://doi.org/10.1155/2014/206370
14. Han, J., Kim, D., Lee, H., Point defects and thermal conductivity in MgO, *Phys. Rev. B* 2023, **108**, 184306. https://doi.org/10.1103/PhysRevB.108.184306
15. de Koker, N., Thermal conductivity of MgO periclase from equilibrium first-principles molecular dynamics, *Phys. Rev. Lett.* 2009, **103**(12), 125902. https://doi.org/10.1103/PhysRevLett.103.125902

16. Mei, Z. G., Shang, S. L., Wang, Y., Liu, Z. K., First-principles study of lattice thermal conductivity of dielectric crystals, *J. Appl. Phys.* 2006, **100**, 023533. https://doi.org/10.1063/1.2216659
17. de Koker, N., Stixrude, L., Karki, B. B., Thermal conductivity of lower mantle minerals, Phys. Rev. Lett. 2009, 103, 125902. https://doi.org/10.1103/PhysRevLett.103.125902
18. Stackhouse, S., Brodholt, J. P., Price, G. D., High-temperature thermal conductivity of MgO, Phys. Rev. Lett. 2010, 104, 208501. https://doi.org/10.1103/PhysRevLett.104.208501
19. Tang, X., Wang, Y., Kuang, Y., Thermal conductivity of Earth's mantle materials, Proc. Natl. Acad. Sci. USA 2010, 107, 4539–4543. https://doi.org/10.1073/pnas.1000395107
20. Dekura, H., Tsuchiya, T., Tsuchiya, J., First-principles study on MgO thermal transport, Phys. Rev. B 2017, 95, 184303. https://doi.org/10.1103/PhysRevB.95.184303
21. Tse, J. S., Thermal conductivity of MgO at high pressure and temperature, J. Phys. Chem. C 2018, 122, 10682–10688. https://doi.org/10.1021/acs.jpcc.8b02916
22. Kwon, S., Lee, S., Lee, S., Influence of defects on thermal properties of MgO, Phys. Rev. B 2020, 102, 184309. https://doi.org/10.1103/PhysRevB.102.184309
23. Han, J., Kim, D., Lee, H., Point defects and thermal conductivity in MgO, Phys. Rev. B 2023, 108, 184306. https://doi.org/10.1103/PhysRevB.108.184306
